# Role of synthetic process parameters of nano-sized cobalt/nickel oxide in controlling their structural characteristics and electrochemical energy performance as supercapacitor electrodes

**DOI:** 10.1038/s41598-024-77180-5

**Published:** 2024-11-08

**Authors:** Marwa Adel, Dina Hassan, Marwa A. A. Mohamed, Taher Salah Edin Kassem, Howida Abouel Fetouh, Sara. E. AbdElhafez, Jehan El Nady

**Affiliations:** 1 Fabrication Technology Department, Advanced Technology and New Materials Research Institute, City of Scientific Research and Technological Applications (SRTA-City), New Borg El-Arab, Alexandria 21934 Egypt; 2 Petroleum Applications Department, Egyptian Petroleum Research Institute (EPRI), Nasr City, Cairo 11727 Egypt; 3 Department of Chemistry, Faculty of Science, Alexandria University, Alexandria, Egypt; 4 Electronic Materials Department, Advanced Technology and New Materials Research Institute, City of Scientific Research and Technological Applications (SRTA-City), New Borg El-Arab, Alexandria 21934 Egypt

**Keywords:** Nano-sized cobalt-nickel oxide, Co-precipitation/hydrothermal synthesis, Recrystallization, Electrochemical energy storage, Supercapacitors, Chemistry, Materials science, Nanoscience and technology, Physics

## Abstract

The synthesis of nano-sized bimetallic Cobalt/Nickel oxides (Ni_1.5_Co_1.5_O_4_) with a 1:1 Co/Ni atomic ratio has been achieved using a surfactant-free co-precipitation/hydrothermal process. The growth mechanism of Cobalt/Nickel oxides Ni_1.5_Co_1.5_O_4_ is elucidated by tuning the synthesis process parameters, including co-precipitation pH and hydrothermal time. The formation of Cobalt/Nickel oxides Ni_1.5_Co_1.5_O_4_ oxide began with the nucleation of cobalt nickel hydroxide nanoplates through the co-precipitation process, followed by dissolution-recrystallization, stacked hexagonal nano-flakes, and a flower-like microstructure. The electrochemical performances of the oxides were evaluated, with the largest surface area observed at pH 9 being the main factor for the best super-capacitive performance. As hydrothermal time increased, the structural directing growth forward, resulted in the formation of a nano-flower structure with a larger surface area. The as-prepared cobalt nickel oxide exhibited a maximum specific capacitance value of 525.5 F g^-1^ at a current density of 1 A g^-1^ and energy and power densities of 88.2 WhKg^-1^ and 606 WKg^-1^, respectively.

**Supplementary Information:**

The online version contains supplementary material available at 10.1038/s41598-024-77180-5.

## Introduction

With the exhaustion of fossil fuels and the increasing environmental pollution, developing clean electrical energy from renewable energy resources is important for the survival of the worldwide community. One of the key issues for the broad application of renewable energies such as solar energy and wind energy is the energy storage for these intermittent renewable energies. Energy storage devices with high-performance materials or advanced technologies have been widely reported in recent years^1^. Supercapacitors have attracted giant interest, as one of the advanced energy storage devices because of their unique functional performance, such as high-power density, fast charge–discharge rates, and good cycling life span, which makes it supplies a clean and efficient way to solve the global energy and environmental crisis^2,3^. Furthermore, supercapacitors can fill the energy and power gaps between conventional electric double-layer capacitors and batteries. Therefore, these devices have started playing vital roles as energy and uninterruptable power sources in electric, fuel cell vehicles, memory backup equipment, cellular phones, and entertainment instruments^4,5^.

Based on the charge storage mechanisms, electrochemical supercapacitors are classified broadly into electrical double-layer capacitors (EDLCs) and pseudo-capacitors. EDLCs, which allow a typical capacitance value of 10–40 μF cm^-2^, rely on electrostatic charge separation between electrolyte ions and electrodes whereas pseudo capacitors demand reversible Faradaic redox reactions on the surface of an electro-active material for charge storage, which can have capacitance value of 10–100 times that of EDLCs^6,7^. Transition metal oxides^8–10^ and hydroxides^11,12^ are the prominent pseudo-capacitive materials. The attention to metal oxides increased considerably during the past several years owing to their high theoretical capacity and promising use as electrode materials for electrochemical energy devices. Many of the conventional and low-cost transition metal oxides or hydroxides have been intensively explored with the target of realizing a high specific capacitance, good rate capability, and long cycle life. V_2_O_5_^13^, NiO^14–18^, MoO_3_^19^, Co_3_O_4_^20^, and MnO_2_^21,22^ have been reported to be highly active as suitable electrode materials. Among these oxides Nickel oxide, has been considered one of the most promising electrode materials for supercapacitor applications due to its satisfactory performance, low cost, natural abundance, high surface area, good redox and charge storage property, controllable size, shape and structural characteristics^14–16^. However, its low electrical conductivity and poor reversibility can worsen the battery’s performance. To date, noteworthy efforts have been made to enhance the electrochemical performance of the electrode materials by combining different electroactive materials. Accordingly, mixed transition metal hydroxides/oxides have received a lot of attention. To further enhance the capacitive performance of nickel hydroxide/oxide, the addition of cobalt into the Ni(OH)_2_/NiO can intensify the electrical conductivity of the final nickel cobalt hydroxides/oxides^23,24^. Moreover, cobalt hydroxide/oxide has been widely reported as a good pseudocapacitive material exhibiting high specific capacitance^11,25,26^. Consequently, the well-designed nickel cobalt double hydroxides/oxides are predicted to possess improved specific capacities with high rate performance and long cycling life^27–33^. Furthermore, forming a three-dimensional (3D) porous architecture and reducing the particle size is worth considering two common varieties, which can synergistically develop the capacitive performance of such binary-metal oxide as a supercapacitor electrode.

Zhang. J. et al.^34^ reported highly porous films composed of Ni-Co binary oxides nanosheets by hydrothermal co-deposition method with hexamethylenetetramine as the nanostructure growth assisting agent. Electrochemical tests demonstrate that binary metal oxides exhibit better electrochemical properties than monometallic oxides. Nevertheless, the high dosage of expensive soft templates and the multifarious post-treatment to eliminate these templates will also frontier the high-yield synthesis of these nickel cobalt hydroxides for applied application. Therefore, developing a template-free method to obtain well-defined 3-D structured nickel cobalt hydroxides/oxides and fine-tuning their morphology, size, and structure is fascinating work. Xiao J. et al.^35^ have established a sea urchin-like bimetallic NiCo_2_O_4_ using urea as a precipitating agent by employing a surfactant-free hydrothermal method for 48 h. These NiCo_2_O_4_ nanomaterials have demonstrated high specific capacitances of 658 F g^-1^ at a current density of 1 A g^-1^. Herein, we report a facile template and surfactant-free co-precipitation/hydrothermal synthesis of nanoflower Cobalt/Nickel oxides Ni_1.5_Co_1.5_O_4_ using ammonium hydroxide (NH_4_OH) as a precipitating agent, employing mild and easier synthetic conditions (time, temperature, pH) as compared with others’ previous work. The oxides’ electrochemical performances have been evaluated. The strategy of growing mechanism and forming the nanostructures is controlled by the co-precipitation pH and hydrothermal time. Deep discussion and interpretation have been presented related to the synthesis conditions, structure, and electrochemical performance. Our findings demonstrate that the supercapacitive performance of as-prepared binary metal oxide is significantly reliant on synthetic process parameters. Furthermore, the porous characteristics of the oxides are significantly allied to their synthetic parameters and also have a major impact on the electrochemical performance.

## Material and methods

### Materials

Cobalt (II) nitrate hexahydrate (Co(NO_3_)_2_·6H_2_O) (97%) and Nickel (II) nitrate hexahydrate (Ni(NO_3_)_2_·6H_2_O) (96%) were obtained from LOBA CHEMIE PVT.LTD., India and Chem-Lab NV, Belgium, respectively. Ammonium hydroxide (NH_4_OH, 33% NH_3_) was obtained from Sigma-Aldrich (Lyon, France). Chemical reagents are analytical grades and were used as received without further purification. Doubly distilled water was used throughout the experiments.

### Synthesis of cobalt/nickel oxide Ni_1.5_Co_1.5_O_4_

Synthesis of Cobalt/Nickel oxides Ni_1.5_Co_1.5_O_4_ involves the preparation of cobalt/nickel hydroxide followed by its calcination. The nickel/cobalt hydroxide was prepared by a facile double-step chemical synthesis route, involving a) co-precipitation in aqueous solution using ammonia as a precipitating agent followed by b) hydrothermal treatment under mild conditions for the dissolution-recrystallization of hydroxides.

#### Effect of the synthesis process co-precipitation pH

In a typical procedure, a total of 60 mL aqueous solutions of 85 mM Cobalt (II) nitrate hexahydrate (Co(NO_3_)_2_·6H_2_O) and 85 mM Nickel (II) nitrate hexahydrate (Ni(NO_3_)_2_·6H_2_O) were added into a beaker. The designed amount of Cobalt/Nickel solutions according to the Co/Ni molar ratio in the final hydroxides was fixed at 1:1. A Few mL of ammonia solution were added dropwise with vigorous stirring**.** Different amounts of added ammonium hydroxide solution through the co-precipitation process have been investigated, including 1 mL, 2 mL, 5 mL, and 10 mL. The pH value of the final reaction solution after an hour of continuous stirring has reached 8, 9, 10, and 10.5, respectively. Afterward, 60 mL of the aqueous suspension was transferred into a 120 mL Teflon-lined autoclave system, heated at 120 °C for 4 h, and then slowly cooled to room temperature. A pale green precipitate was formed in the Teflon tube through the hydrothermal process. It was filtered, washed several times with distilled water, and then dried in a vacuum oven at 70 °C for 24 h. The samples are denoted as NiCo(OH).1mL, NiCo(OH).2mL, NiCo(OH).5mL and NiCo(OH).10mL, respectively. Finally, the dried precipitate was calcinated at 300 °C for 2 h in the air to obtain the final black oxide sample. The samples are denoted as NiCo(O).1mL, NiCo(O).2mL, NiCo(O).5mL, and NiCo(O).10mL, respectively.

#### Effect of the synthesis process hydrothermal time

The optimum pH value of the co-precipitation reaction, regarding the morphological features, structural quality, and electrochemical performance, has been determined. Later on, different hydrothermal process times were studied, including 4 h, 6 h, 8 h, 10 h and 12 h. The produced samples are symbolized as NiCo(OH).4h, NiCo(OH).6h, NiCo(OH).8h, NiCo(OH).10h and NiCo(OH).12h, respectively. Lastly, the corresponding obtained black oxide samples from the calcination step are designed as NiCo(O).4h, NiCo(O).6h, NiCo(O).8h, NiCo(O).10h and NiCo(O).12h, respectively.

### Characterizations

X-ray diffraction (XRD) was used to identify the produced powder samples and characterize their crystallographic structure. XRD patterns were obtained using PANalytical’s X’Pert PRO MRD diffractometer (40 kV, 40 mA, Cu Kα 1 Ni-filtered radiation, λ = 0.15406 nm; Malvern Panalytical Ltd Corporation, Malvern, United Kingdom). The analysis was performed over a 2θ range of 4–90°, at a scanning step time of 0.7 s and a scanning step of 0.02°. The morphology was analyzed by a scanning electron microscope (SEM, Model JSM 6010 LV; JEOL Ltd., Tokyo, Japan) and a high-resolution transmission electron microscope (HR-TEM, JEOL JEM-2100; JEOL Ltd.), operating at 20 and 200 kV, respectively. SEM images were captured for the solid powder. HR-TEM samples were prepared by dropping a colloidal suspension of the powder sample in ethanol, on a carbon-coated copper grid and letting the solvent to evaporate. Furthermore, the purity and composition of nickel cobalt oxides were also measured by EDX (energy dispersive spectrometer Model JSM 6360 LA; JEOL Ltd., Tokyo, Japan).

### Electrochemical measurements

To prepare the working electrode, the prepared active material NiCo(O) (90 wt%), carbon black (5 wt %), polyvinylidene fluoride PVDF (5 wt %) as a binder, and 1-methyl 2-pyrrolidinone as a solvent were mixed to form a slurry. The slurry was then doctor-bladed onto a nickel sheet (1 cm^2^) and dried at 60 °C overnight. The mass of active material in the active surface was about 1 to 1.5 mg cm^−2^.

The electrochemical measurements were performed in a three-configuration electrode cell using Ag/AgCl as the reference electrode and platinum as the counter electrode. Sodium sulfate (Na_2_SO_4_ 3M) is used as an electrolyte_._ The measurements were performed at room temperature using a computer-controlled Poteniostate (Metr ohm Auto lab, model: 87070) involving cyclic voltammetry (CV), galvanostatic charge/discharge (GCD), and electrochemical impedance spectroscopy (EIS).

The CV studies were performed between 0 to 1 V at increasing sweep rates from 5 mV /s to 100 mV/S. The charge-discharge measurements were performed at 1A/g. The specific capacitance of supercapacitor (C) can be calculated by the Eq. 1^36,37^:

1 $$C = { }\frac{{{\text{I}} \times \Delta {\text{t}}}}{{{\text{m}} \times \Delta {\text{V}}}}$$

where I, ∆t, ∆V, and m are the discharge current (A), discharge time (s), potential window (V), and the mass of the active material (g), respectively.

Energy density (E) and power density (P) were calculated using the Eq. 2&3, respectively^36,37^:

2 $$E = \frac{{{\text{C}} \times \Delta V^{2} }}{2 \times 3.6}$$

3 $$P = \frac{{{\text{E}} \times 3600}}{\Delta t}$$

## Results and discussions

### Effect of the synthesis process co-precipitation pH

#### Microstructure of the synthesized Ni/Co oxide samples

Material identification, composition and crystal structure

*EDX* Analyses have been carried out on the synthesized metal oxide samples at different added amounts of ammonia and, as a consequence, variant reaction solution pH, to explore the real Ni/Co atomic ratio within samples. The samples consist of Ni, Co, and O. The real Ni/Coatomic ratio was found to be 0.97:1, 0.93:1, 0.62:1, and 0.53:1 for the NiCo(O).1mL, NiCo(O).2mL, NiCo(O).5mL, and NiCo(O).10mL samples, respectively. It is clear that as the added amount of ammonia/pH value is increased the real ratio deviates from the designed one. Thus, the real Ni/Co atomic ratio of NiCo(O).1mL and NiCo(O).2mL are very close to the designed value (1:1) during synthesis, demonstrating that the Ni^2+^ and Co^2+^ ions were completely precipitated in the Co-precipitation/hydrothermal process. However, the synthesized samples with high concentrations of ammonia show divergent behavior. This can be attributed to the higher tendency and affinity of cobalt ions to co-precipitate faster than the nickel ions^35,38^ Accordingly, NiCo(O).1mL and NiCo(O).2mL are considered the most favorable samples among the synthesized oxide samples at different added amount of ammonia/pH value. Table S1 in supplementary materials. Indicates the atomic and mass contents of Co, Ni, and O elements within the nickel cobalt oxides samples, estimated by EDX analysis. EDX spectra are included as Supplementary Figure S1.

*XRD analysis* XRD spectra of the co-precipitated Nickel cobalt hydroxide samples at different pH values (NiCo(OH).1mL, NiCo(OH).2mL, NiCo(OH).5mL and NiCo(OH).10mL) are shown in Supplementary Figure S2a, b, c and d, respectively. It is well known that, both nickel hydroxide and cobalt hydroxide have two polymorphs with hexagonal-layered structure, namely α and β phase, respectively^39,40^. The two polymorphs are different in the stacking of these layers. Layers can be well ordered and closely packed along the c-axis as in the β-phase, which is characterized by stoichiometric composition and interlayer separation of 4.6 Å or could be randomly stacked along the c-axis with water or anionic intercalates residing within the Van der Waals gap as in the α-phase which is hydroxyl-deficient phase containing interlayer anions. Intercalating species result in a larger interlayer separation (7–8 Å) depending on the size and bonding of the intercalating species^40,41^. When ammonia is used as the precipitation agent, anions (NO^-3^) and small molecules (NH_3_, H_2_O) can be embedded in the interlayer structure to form some α-phase. After the hydrothermal process, dissolution-recrystallization favors the formation of the stoichiometric β-phase^39^. The diffraction patterns of NiCo(OH).1mL, NiCo(OH).2mL and NiCo(OH).5mL, which were synthesized by adding 1mL, 2mL and 5mL of ammonia, demonstrate peaks at 2θ = 19.1°, 32.7°, 38.2°, 51.7°, 58.5°and 62.08° with d-spacing 4.62, 2.73, 2.35, 1.76, 1.57 and 1.49 Å, corresponding to the (001), (100), (002), (021), (003)and (111) planes, respectively, which can be indexed exactly to the β phase^34,41–43^, (JCPDS 1074-1057).

The peaks assigned to α(001), α(002) and α(110), are also observed at about 11.2°, 22.73° and 34.6° with d-spacing 7.86, 3.91 and 2.592 Å, respectively. These results reveal the co-existence of α and β phases^39^ in the NiCo(OH).1mL, NiCo(OH).2mL and NiCo(OH).5mL. The peaks located at high angles (69.7°, 72.12^o^ and 81.75°) can be attributed to the high index facets of β-phase nickel hydroxide^44^, which are exclusively observed in the spectrum of NiCo(OH).1mL, as well as NiCo(OH).2mL, in Supplementary Figure S2a, b, respectively and become trivial in the spectrum of NiCo(OH).5mL, as shown in Supplementary Figure S2c. This is consistent with the EDX result, which depicts the decrease of the Ni content as the added amount of ammonia increased. Nevertheless, with the further increase of the added amount of ammonia up to 10mL, these high-angle peaks vanished. Moreover, the spectrum of NiCo(OH).10mL in Supplementary Figure S2d, shows five additional diffraction bands located at 31.01°, 36.5°, 44.5°, 55.26° and 64.7° which indexed to the cobalt hydroxide Co(OH)_2_ (JCPDS 30-0443)^34,39^. This confirms the same result obtained from the EDX analysis of the binary oxides, as the complete conversion of the binary metal hydroxides (NiCo(OH)_2_) to their corresponding metal oxides (NiCo_2_(O)_4_) and released water vapors during the calcination process. Hence, their constituents have the same ratio. This indicates the significantly higher content of Co to Ni for synthesized samples at a higher added amount of ammonia. The higher tendency and affinity of the cobalt ions to co-precipitate faster than the nickel ions could be attributed to the smaller solubility product constant (K_sp_) at 25 °C of Co (OH)_2_ (2.5 × 10^–16^) than those of the Ni(OH)_2_ (2.8 × 10^–16^)^35^. The possible mechanism can illuminate the reason for this behavior. Briefly through the hydrothermal process, sluggish hydrolysis of ammonia engenders OH^-^ ions within the reaction medium which inducts coordinate with Ni ^+2^ and Co^+2^ cations to form different phases of Ni(OH)_2_ and Co(OH)_2_. The creation of the OH^-^ ions takes place at different added amounts of ammonia, which leads to different precipitation reaction rates. This finally further manipulates the crystal growth process.

Furthermore, the more intense and sharp diffraction patterns demonstrate the high degree of crystallinity of the two samples NiCo(OH).1mL, NiCo(OH).2mL that have been prepared at low amounts of ammonia/pH value. The average crystallite size of β phase, perpendicular to the(001) plane, (D001) has been calculated using Sherrer’s equation^45^, which is expressed by Eq. (4):

4 $${\text{D}}\left( {00{1}} \right) = {\text{Kl}}/{\text{Bcosq}}$$

where K is a constant dependent on the crystallite shape (0.89), λ is the x-ray wavelength, B is the full width at half maximum, and θ is the scattering angle of the (001) peak. The average crystallite size is found to be 80.96, 57.81, 50.6, and 33.7 nm for NiCo(OH).1mL, NiCo(OH).2mL, NiCo(OH).5mL and NiCo(OH).10mL, respectively. Whilst, the average crystallite size of α phase, was found to be 34.3, 26.74, 23.6, and 12.5 nm for NiCo(OH).1mL, NiCo(OH).2mL, NiCo(OH).5mL and NiCo(OH).10mL, respectively. The calculated average crystallite size of both phases and their intensity ratios, which are obtained from the instrument software are listed in Table 1 for all samples synthesized at different added amounts of ammonia/different pH values. There is a remarkable decrease in the calculated average crystallite size of both two phases as the added amount of ammonia/pH values increased. This could be attributed to the rapid increase of the reaction rate as the pH value of the reaction raised. Thus, the crystallites have no sufficient time to enlarge in size.

**Table 1:** The average crystallite size of α and β phases of NiCo(OH) samples prepared at different added amount of ammonia/different pH values and their intensity ratios.

Samples codes	D _β_ (nm)	D _α_ (nm)	I_β_%	I_α_%	I_α_/I_β_
NiCo(OH).1mL	80.9	34.3	100	4.7	0.047
NiCo(OH).2mL	57.8	26.7	100	8.3	0.083
NiCo(OH).5mL	50.6	23.6	100	58.7	0.587
NiCo(OH).10mL	33.7	12.5	69.6	32.4	0.466

Moreover, the increase of Ammonia/ pH value up to 5 mL and 10 mL results in a decrease in the sharpness of the diffraction patterns which means a decrease in the degree of crystallinity. The co-existence of the two phases is still present in the higher added amount of ammonia as well as the lower ones. However, the ratio of the two phases shows a variation by increasing the amount of ammonia. The ratio of the deficient phase, α phase, increased gradually as the amount of Ammonia/pH value increased as depicted in the tabulated data in Table 1. All these notifications with two advantages; i) a higher degree of crystallinity and ii) the majority of the more perfect crystalline phase, point to the preferred lower pH value/ amount of ammonia during the co-precipitation process.

Finally, XRD illustrates that NiCo(OH).1mL and NiCo(OH).2mL are the optimum samples regarding the highest degree of crystallinity, and the lowest ratio of the deficient phase which means highest degree of perfection.

**Fig. 1:**
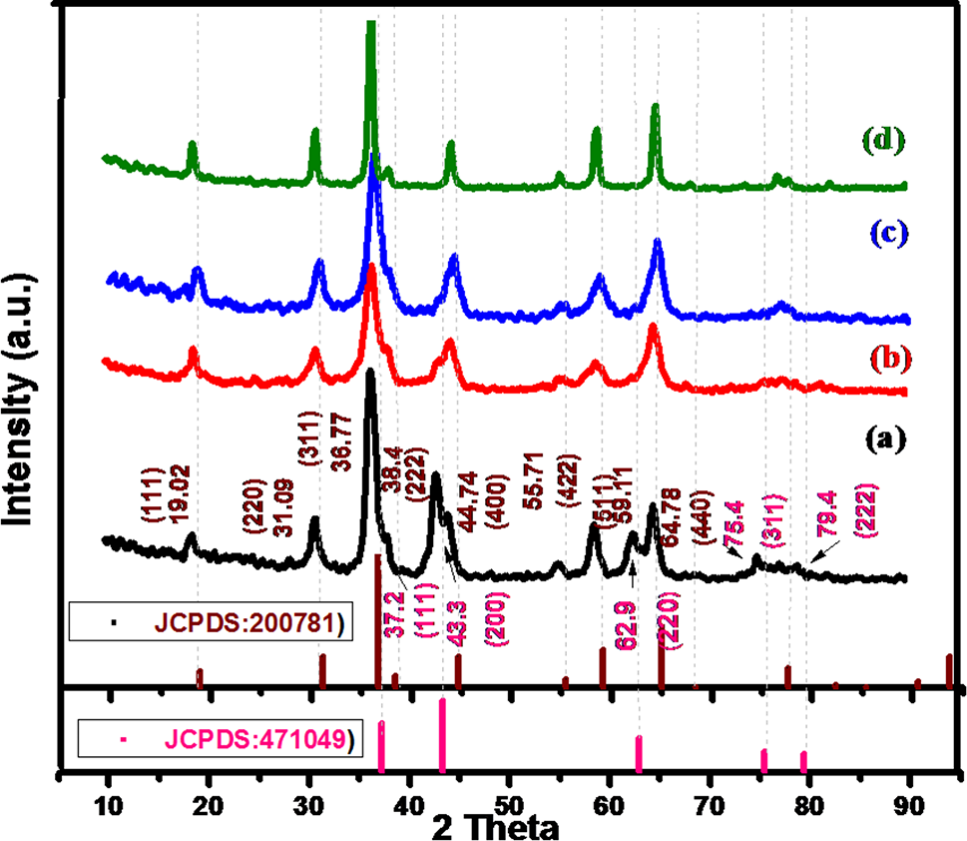
The XRD spectra of synthesized Nickel cobalt oxide samples at different added amount of ammonia/different pH values (**a**) NiCo(O).1mL, (**b**) NiCo(O).2mL, (**c**) NiCo(O).5mL and (**d**) NiCo(O).10mL.

The XRD spectra of Nickel cobalt oxide samples NiCo(O).1mL, NiCo(O).2mL, NiCo(O).5mL and NiCo(O).10mL are shown in Fig. 1. The detected eight diffraction peaks at 2θ of 19.02°(111), 31.09°(220), 36.7°(311), 38.4°(222), 44.7°(400), 55.7°(422), 59.1°(511) and 64.7° (440) can be indexed to Ni_1.5_Co_1.5_O_4_ (JCPDS No. 20-0781). There are also five visible diffraction peaks, located at 2θ of 37.2°(111), 43.3°(200), 62.9°(220), 75.4°(311), and 79.4°(222), which can be indexed to NiO (JCPDS. No. 47-1049)^42^. No other peaks from hydroxides can be observed. That means a complete transition from hydroxide to oxide after annealing at 300 °C. The diffraction peaks of NiCo(O) are broader than those of NiCo(OH), implying a smaller crystal size of the former than the latter. The average crystallite size, perpendicular to the (311) plane, (D311) is found to be 11.67, 11.68, 26.29, and 35.05 nm for NiCo(O).1mL, NiCo(O).2mL, NiCo(O).5mL and NiCo(O).10mL, respectively. We hypothesize that the binary oxide is composed of Ni_1.5_Co_1.5_O_4_ and a small portion of NiO by synthesis at the low amount of ammonia. As the added amount of ammonia increased to 10mL in NiCo(O).10mL the detected diffraction peaks of NiO diminished. This agrees with the EDX results which depict the decrease of nickel fraction compared with the one of cobalt in the synthesized samples with increasing the amount of ammonia during synthesis up to 10 mL.

Morphology

*SEM* The morphologies of both Nickel Cobalt Hydroxides and their oxides were directly examined by scanning electron microscopy (SEM). The micrographs of NiCo(OH).1mL, NiCo(OH).2mL, NiCo(OH).5mL and NiCo(OH).10mL with their magnifications are shown in Supplementary Figure S3. Plenty of methods have been utilized to synthesize Ni/Co (hydroxide and oxide) with different morphological architectures including nanoparticles, nanoflakes, and nanoflowers^35,42,43,46^. Herein, The SEM images of NiCo(OH).1mL and NiCo(OH).2mL samples, which have been synthesized by adding 1and 2 mL ammonia into reaction solution making pH value of the co-precipitation solution 8 and 9, respectively, exhibit sheet-like/nanoplates-like architectures with irregular hexagonal ring, as shown in Supplementary Figures S3a, b and S3c, d, respectively. Their thickness is < 100 nm, ~ 95 nm. Locally these nanoplates are arranged parallel to each other. Whilst, we observe an aggregation by increasing the amount of ammonia up to (5 mL/ pH ~ 10) in NiCo(OH).5mL Supplementary Figure S3e, f. This aggregation is intensified and become more bulky by further increase of the amount of added ammonia up to 10 mL/ pH ~ 10.5 in NiCo(OH).10mL Supplementary Figure S3g, h. Furthermore, incomplete growth of the hexagonal rings was observed in NiCo(OH).5mL and NiCo(OH).10mL.

This could imply that there is a competitive effect between the two roles of OH^-^ ions that control the reaction rate and play a role in the formation of the binary metal hydroxide nanosheets: i) an increase in the reaction rate of crystal growth formation and ii) micelles formation, which act as a capping agent for the metal ions surface. Firstly, by small increase of pH value from 8 to 9 by increasing the amount of added ammonia from 1 to 2 mL, there is an effective enhancement of the surface passivation of the crystal growth due to the interaction of the metal cation (Ni ^+2^, Co ^+2^) with the hydroxide anions (OH^-^) of the ammonium hydroxide without boasting the precipitation reaction rate. This, in turn, leads to a limited degree of aggregation of nanoplates within NiCo(OH).1mL and NiCo(OH).2mL. However, beyond pH 9, the precipitation reaction rate is accelerated which intensifies the aggregation of nanoplatelets. Thus, it leads to a reduction of the surface area of the obtained NiCo hydroxides in NiCo(OH).5mL and NiCo(OH).10mL.

**Fig. 2:**
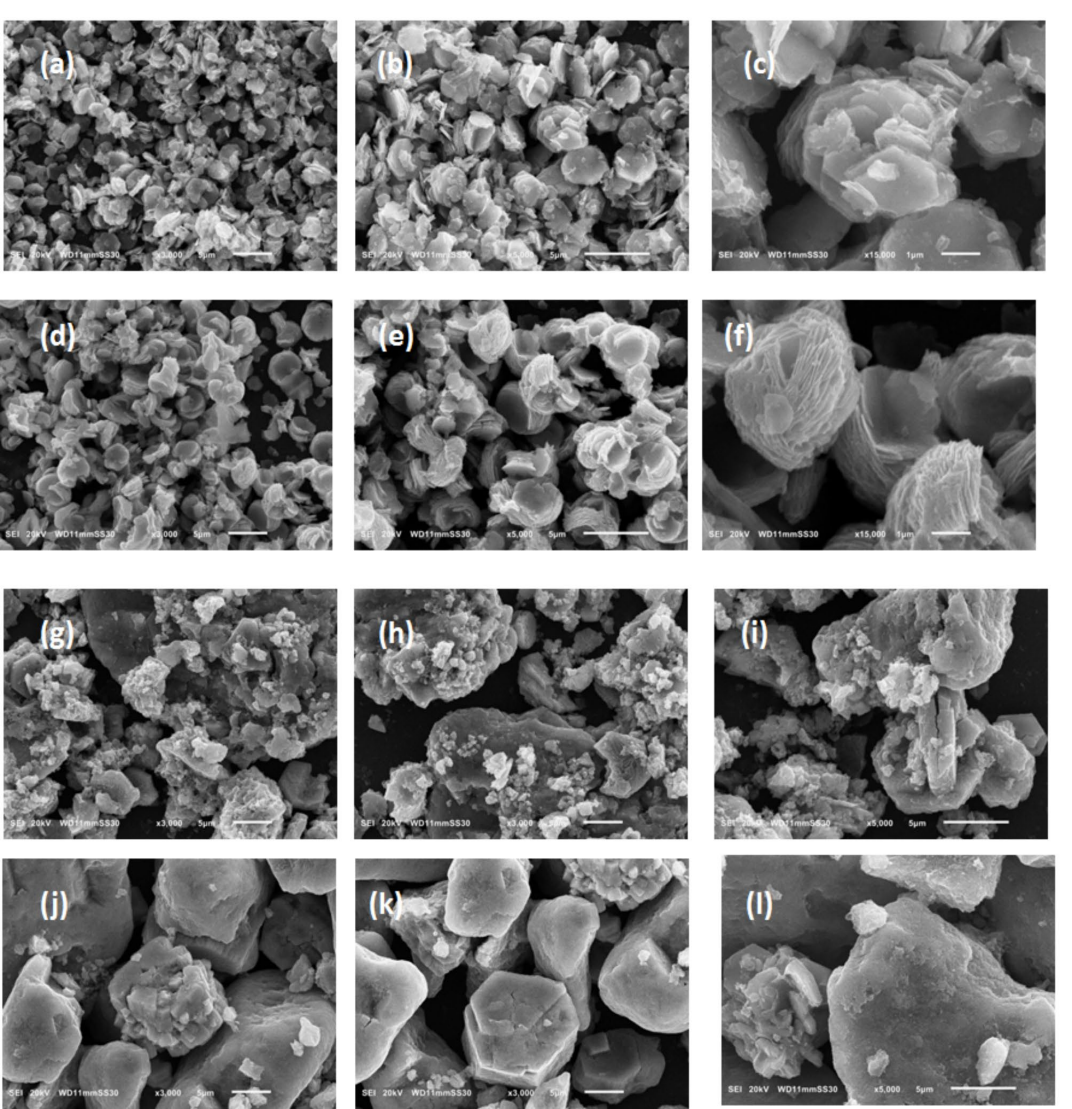
The SEM micrographs of (**a**) NiCo(O).1mL, (**b**, **c**) Magnified micrographs of NiCo(O).1mL, (**d**) NiCo(O).2mL, (**e**, **f**) Magnified micrographs of NiCo(O).2mL, (**g**) NiCo(O).5mL, (**h**, **i**) Magnified micrographs of NiCo(O).5mL, (**j**) NiCo(O).10mL and (**k**, **l**) Magnified micrographs of NiCo(O).10mL.

Regarding the Nickel Cobalt oxide samples, the micrographs of NiCo(O).1mL and NiCo(O).2mL samples are shown in Fig. 2a, d, we can see that the morphology is nearly conserved during the phase transformation from the metal hydroxide to the corresponding metal oxide. Interestingly, the average lateral dimension of the oxide nanosheets is almost conserved, ~ 3.3 and 3.7 μm for NiCo(O).1mL and NiCo(O).2mL, respectively. Whilst the oxide nanoplates become thinner, ~ 77 nm and considerably separated/exfoliated as shown in the magnified micrographs of the NiCo(O).1mL and NiCo(O).2mL in Fig. 2b, c and Fig. 2e, f, respectively. This is attributed to the released water vapors (H_2_O) during the calcination process in conversion of binary metal hydroxides (NiCo(OH)_2_) to their corresponding metal oxides (NiCo_2_(O)_4_).This leads to more excellent exfoliation and gives an explanation for the decreased thickness. At the same time as, the aggregation is still observed in NiCo(O).5mL and NiCo(O).10mL in Fig. 2g, j, h and k, and becomes more intensified and bulky in NiCo(O).10mL, the magnified micrographs Fig. 2l than NiCo(O).5mL as declared in the magnified micrographs in Fig. 2i.

Finally, according to the microstructure analysis using EDX, XRD and SEM we conclude that the pH value of the co-precipitation reaction has a significant effect on the microstructure of the prepared hydroxides/oxides samples. The low pH value is preferred to high ones. The sample that synthesized at (low amount added of ammonia 1 mL and 2 mL/ low pH value ~ 8 and 9) NiCo(O).1mL and NiCo(O).2mL have comparable microstructure characteristics. They are considered the optimum samples regarding, (i) the highest degree of the active surface passivation of the crystal growth and (ii) the lowest crystal size formed, and (iii) the lowest limited degree of aggregation. Whilst, NiCo(O).2mL exclusively depicts their nanosheets have (i) highest degree of exfoliation (ii) largest lateral dimension i.e., the highest surface area.

HR-TEM and SAED.

**Fig. 3:**
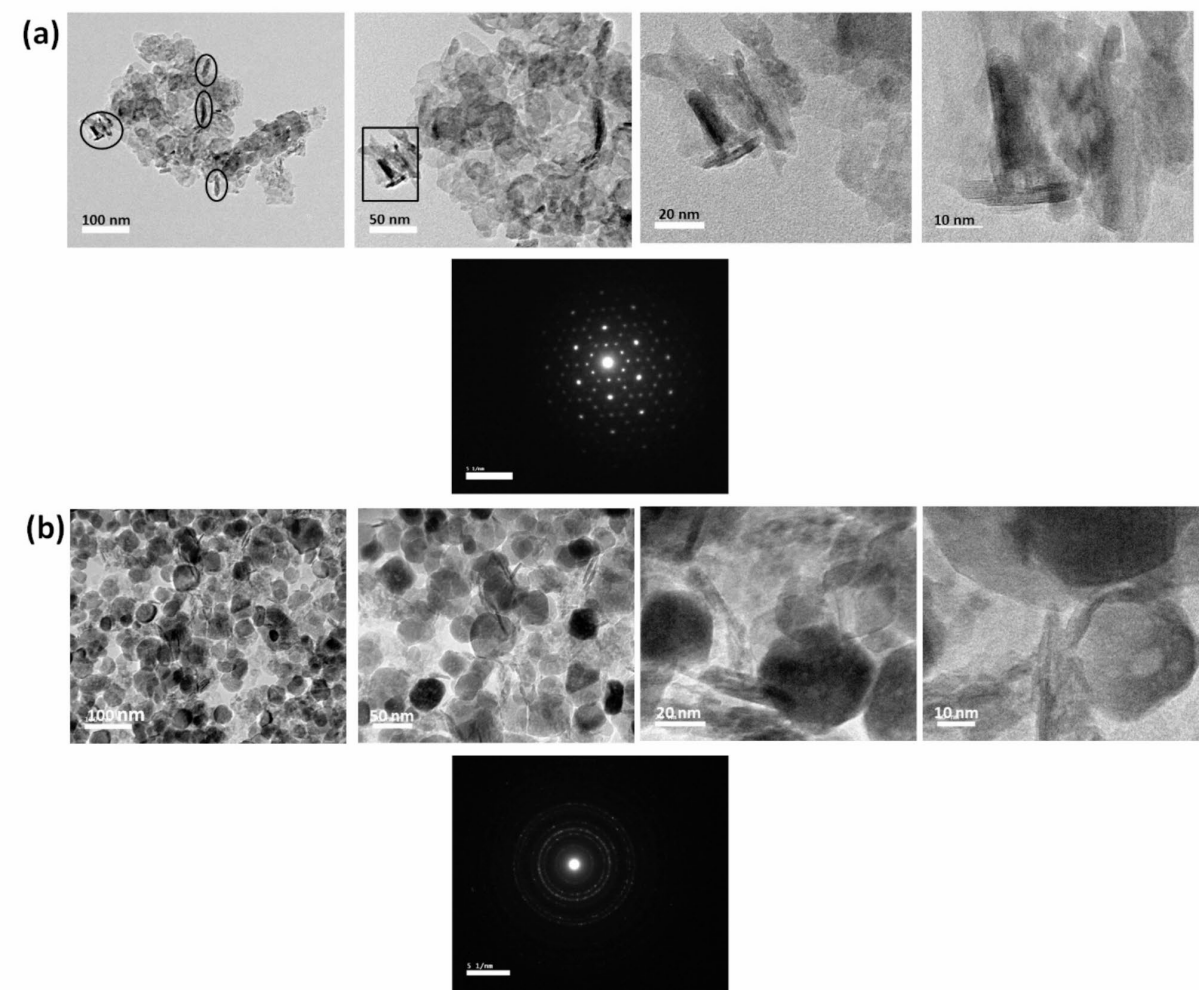
TEM images of (**a**) NiCo(O).2mL, under different magnifications) and the corresponding selected area SAED of NiCo(O).2mL, (**b**) TEM images of NiCo(O).10mL, under different magnifications and the corresponding selected area SAED of NiCo(O).10mL.

Figure 3a presents the morphological and structural features of the NiCo(O).2mL that were synthesized at the low added amount of ammonia/low pH value through TEM and SAED. Figure 3a displays a sheet-like structure. Locally, these sheets are somewhat packed as separated/exfoliated parallel sheets form random oriented bundles (not all in the same plane), and within these there are bundles of standing nanosheets appear as nanorods with length up to ~ 20 nm, as illustrated in Fig. 3a *the two first columns with bar scale, 100 nm and 50 nm* and the magnified micrographs in Fig. 3a *in the 3*^*rd*^* and 4*^*th*^* columns with bar scale 20 nm and 10 nm*, respectively. The diameter of Ni_1.5_Co_1.5_O_4_ is about 50 nm and their thickness are roughly < 1 nm. The phase of the metal oxide nanoparticles was also identified by selected area diffraction SAED technique in the Fig. 3a. The distinct visible, well defined diffraction spots indicate the crystalline nature of the NiCo(O).2mL nanosheets. Figure 3b depicts the morphological and structural features of the NiCo(O).10mL that were synthesized at the highest added amount of ammonia/highest pH value through TEM with their different magnifications and SAED. Moreover, a good agreement with the XRD spectrum has been confirmed, the lattice parameters hkl plan (220), (111) of the XRD of Ni_1.5_Co_1.5_O_4_ and NiO, were well matched with the magnified micrographs of HR-TEM represented in Supplementary Figures S4a and S4b, respectively.

By increasing the added amount of ammonia 10 mL /pH value up to the highest value 10.5, in sample NiCo(O).10mL. The sheets become denser and darker which confirms the aggregation nature of the formed sheets as declared from the SEM results. Besides, there are also observed bundles of standing nanosheets, Fig. 3b, in the 3^rd^ column with bar scale 20 nm, from which we can estimate the thickness of nanosheets was about ~ 5 nm.The samples are in the form of the spots becoming less visible, randomly dotted ring (almost unclear and overlapped ring) in, suggesting that the crystallinity decreases and the polycrystalline nature of NiCo(O).10mL. Finally, the comparison with the X-ray Diffraction (XRD) data ensures the consistency between the crystallographic information obtained from both techniques. The lattice parameters hkl plan (311) of the XRD of Ni_1.5_Co_1.5_O_4_, was well matched with the magnified micrographs of HR-TEM represented in Supplementary Figure S4c.

Finally, according to the microstructure analysis using EDX, XRD, SEM, TEM and SAED we conclude that the sample that synthesized at low amount added of ammonia 2mL/ low pH value ~ 9, NiCo(O).2mL, considered the optimal sample regarding its well microstructure characteristics.

### Effect of the synthesis process hydrothermal time

#### Composition and crystal structure

EDX

Analyses have been carried out on the synthesized metal oxide samples at different hydrothermal time to explore the real Ni/Co atomic ratio within samples. Evidently, the samples consist of Ni, Co and O. The real Ni/Co atomic ratio was found to be 0.93:1, 0.92:1, 0.88:1, 0.94 :1 and 0.99:1 for the NiCo(O).4h, NiCo(O).6h, NiCo(O).8h, NiCo(O).10h and NiCo(O).12h samples, respectively. It was noted the real Ni/Co atomic ratio of all the prepared samples are almost close to the designed value (1:1) during synthesis except NiCo(O).8h. This demonstrates that the Ni^2+^ and Co^2+^ ions were almost completely precipitated in the hydrothermal process. However, the sample that was prepared at hydrothermal time of 8 h shows divergent behavior. Table S2, as a Supplementary material. Indicates the atomic and mass contents of Co, Ni and O elements within the nickel cobalt oxides samples, estimated by EDX analysis. EDX spectra are included as Supplementary Figure S1.

XRD

XRD spectra of Nickel Cobalt hydroxide samples that are synthesized at different hydrothermal time 4, 6, 8, 10 and 12 h are shown in Supplementary Figure S5a, b, c, d and e, respectively. All the peaks assigned to α and β phases of Nickel cobalt hydroxide are observed in all the prepared at different hydrothermal time. The co-exist of the two phases is still observed as the time is elevated. However, the ratio of the two phases shows a variation by increasing the hydrothermal time. The ratio of the deficient phase, α phase recorded a lowest value for Nickel cobalt hydroxide that synthesized at hydrothermal time 10 h, NiCo(OH).10h. Table 2 presents the calculated average crystallite size of both phases and their intensity ratios for all the prepared samples at different hydrothermal time.

**Table 2:** The average crystallite size of α and β phases and their intensity ratios for all the prepared samples at different hydrothermal time.

(Samples code)	D _β_ (nm)	D _α_ (nm)	I_β_%	I_α_%	I_α_/I_β_
NiCo(OH).4h	57.8	26.7	100	8.3	0.083
NiCo(OH).6h	64.4	20.0	100	8.78	0.087
NiCo(OH).8h	50.6	36.4	100	13.0	0.13
NiCo(OH).10h	101.3	9.55	100	8.05	0.080
NiCo(OH).12h	101.2	22.3	100	9.02	0.090

**Fig. 4:**
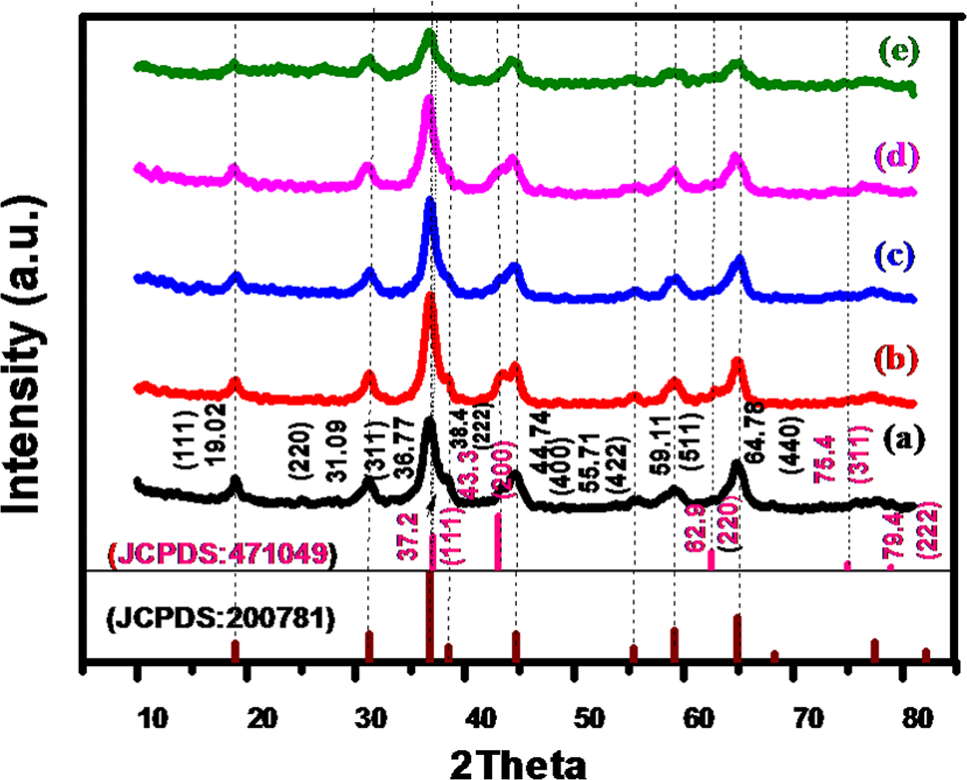
XRD spectra of synthesized Nickel cobalt oxide samples at different hydrothermal time (**a**) (NiCo(O).4h, (**b**) NiCo(O).6h, (**c**) NiCo(O).8h, (**d**) NiCo(O).10h) and (**e**) NiCo(O).12h).

The XRD spectra of Nickel cobalt oxide samples NiCo(O)4h, NiCo(O)6h, NiCo(O)8h, NiCo(O)10h and NiCo(O)12h are shown in Fig. 4. Obviously, all the eight diffraction peaks, which are indexed to Ni_1.5_Co_1.5_O_4_ (JCPDS No. 20–0781), are detected in all the samples as the hydrothermal time upraised. Nevertheless, the feeble peaks of NiO are getting feebler. Finally, they almost could not be detected as the time elevated up to 12 h in NiCo(O)12h. This means that the obtained metal oxide is mainly only Ni_1.5_Co_1.5_O_4_. The average crystallite size, perpendicular to the (311) plane, (D311) is found to be 11.67,8.76, 24.73, 10.51 and 10.51 nm for NiCo(O).4h, NiCo(O).6h, NiCo(O).8h, NiCo(O).10h and NiCo(O).12h, respectively. All have a comparable tens nanometers crystallite size. Finally, we can conclude that increasing the hydrothermal time leads to improve the degree of purity.

#### Morphology

SEM

The morphologies of Nickel Cobalt Hydroxides, which are synthesized at various hydrothermal times, are analyzed using SEM and the micrographs are given in Supplementary Figure S6 of the Supporting Information. The SEM images of NiCo(OH).4h, NiCo(OH).6h, NiCo(OH).8h, NiCo(OH).10h samples, exhibit hexagonal sheet-like/nanoplates-like architectures, their lateral dimension are ~ 3.5, 3.8, 5.5, 5 and 4 µm as shown in Supplementary Figure S6a, b, c, d and e, respectively. As we mentioned above that the pH value of the co-precipitation process plays a main role in avoiding the random agglomeration of the primarily formed crystallites by successfully shielding the OH^-^ activity further on the nascent crystallites. Meanwhile the initially formed Ni Co (OH)_2_ crystallites and their growth define the final morphology. Herein, the hydrothermal time affects the flakes growth and dimensionality. Obviously, increasing the time lets the hexagonal flakes undergo extensive growth in size/dimensionality during the hydrothermal process. It is apparently clear that the lateral dimension gradually increased as the hydrothermal time increased up to 8 h, then it slightly decreased in 10&12 h but still larger than 4&6 h.

**Fig. 5:**
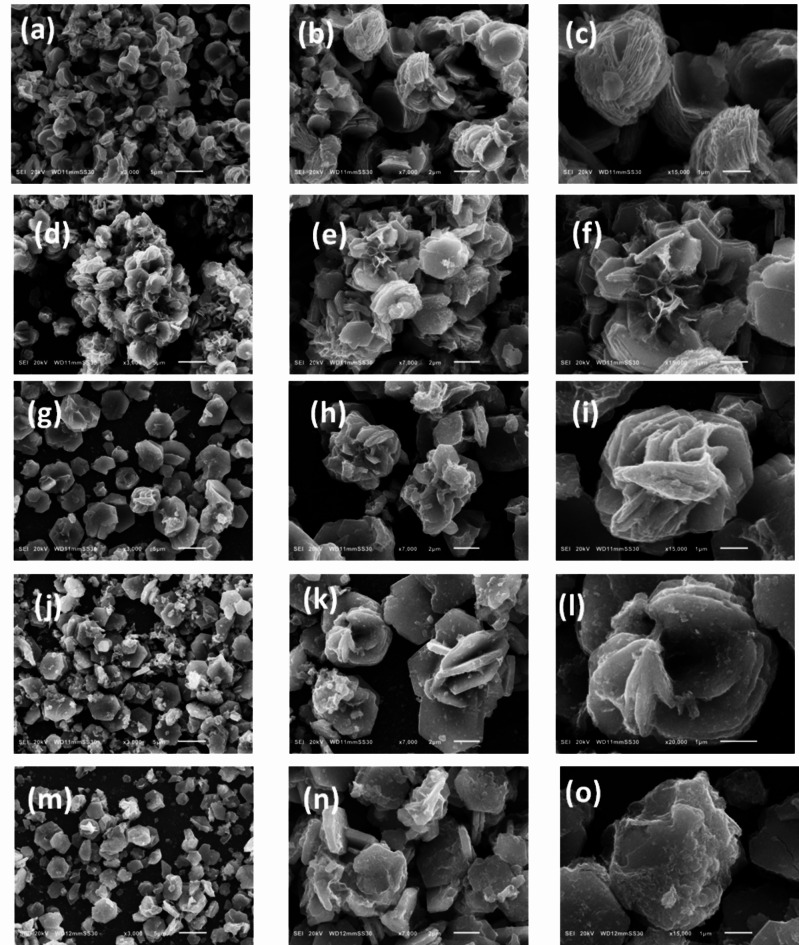
The SEM micrographs of (**a**) NiCo(O).4h, (**b**, **c**) Magnified micrographs of NiCo(O).4h, (**d**) NiCo(O).6h, (**e**, **f**) Magnified micrographs of NiCo(O).6h, (**g**) NiCo(O).8h, (**h**, **i**) Magnified micrographs of NiCo(O).8h, (**j**) NiCo(O).10h, (**k**, **l**) Magnified micrographs of NiCo(O).10h, (**m**) NiCo(O).12h and (**n**, **o**) Magnified micrographs NiCo(O).12h.

Regarding the Nickel Cobalt oxide samples, the micrographs of NiCo(O).4h, NiCo(O).6h, NiCo(O).8h, NiCo(O).10h and NiCo(O).12h with their magnifications are shown in Fig. 5. The calcinied oxide samples show somewhat different morphologies unlike their uncalcinied hydroxide counterparts but with noticeably high degree of porosity. Firstly, the thin 2D-nano-flakes, with comparable lateral dimensions to their hydroxides counterparts, become more exfoliated and arrange them self in a particular assemblage like to semi-flower as shown by comparing the magnified micrographs of NiCo(O)4h and NiCo (O)6h Figs. 5c, f, respectively. Besides, there is significant pore structure, increase of exfoliation and flower shape becomes more apparent in the SEM images of Ni Co O samples as the hydrothermal time gradually increased from 4 h up to 8 h, the magnified images Fig. 5i. Moreover, the morphology of the as prepared NiCo(O)10h sample becomes a complete nano-flower like morphology with 3D-porous structure, the magnified image in Fig. 5l. Lastly, the hexagonal rings still appeared but the nanoflowers didn’t appear as the hydrothermal time increased up to 12 h in NiCo(O)12h, Fig. 5m, n, o. Besides, the sheets become thicker and others bulky have irregular shapes. The reason for this drawback is not clear up to now. This drawback in the morphology of the formed sheets which acquired with increasing the hydrothermal time was also observed and reported by Liang et al^47^. So it is expected, the surface area and porosity of the Ni Co O samples are significantly higher than their respective NiCo(OH)_2_ precursors. The nanoflower architecture that appeared in the oxide sample at NiCo(O)10h with its large thin flakes and its pore nature^34,39,48,49^ let one expect that NiCo(O)10h will be the optimum sample regard the surface area and the electrochemical performance. The effect of time of hydrothermal process on the final morphology is quite immense, the hexagonal flakes start to impinge on the neighboring flakes and assemble along a particular crystal orientation. As the hydrothermal time increases, it forces the impact of structural directing growth forward completing formation of nano-flower structure composed of interconnected nanoplates which again imparts the oxide nanostructure with larger surface area than the case of stack of nanoplates over each other.

HR-TEM and SAED

**Fig. 6:**
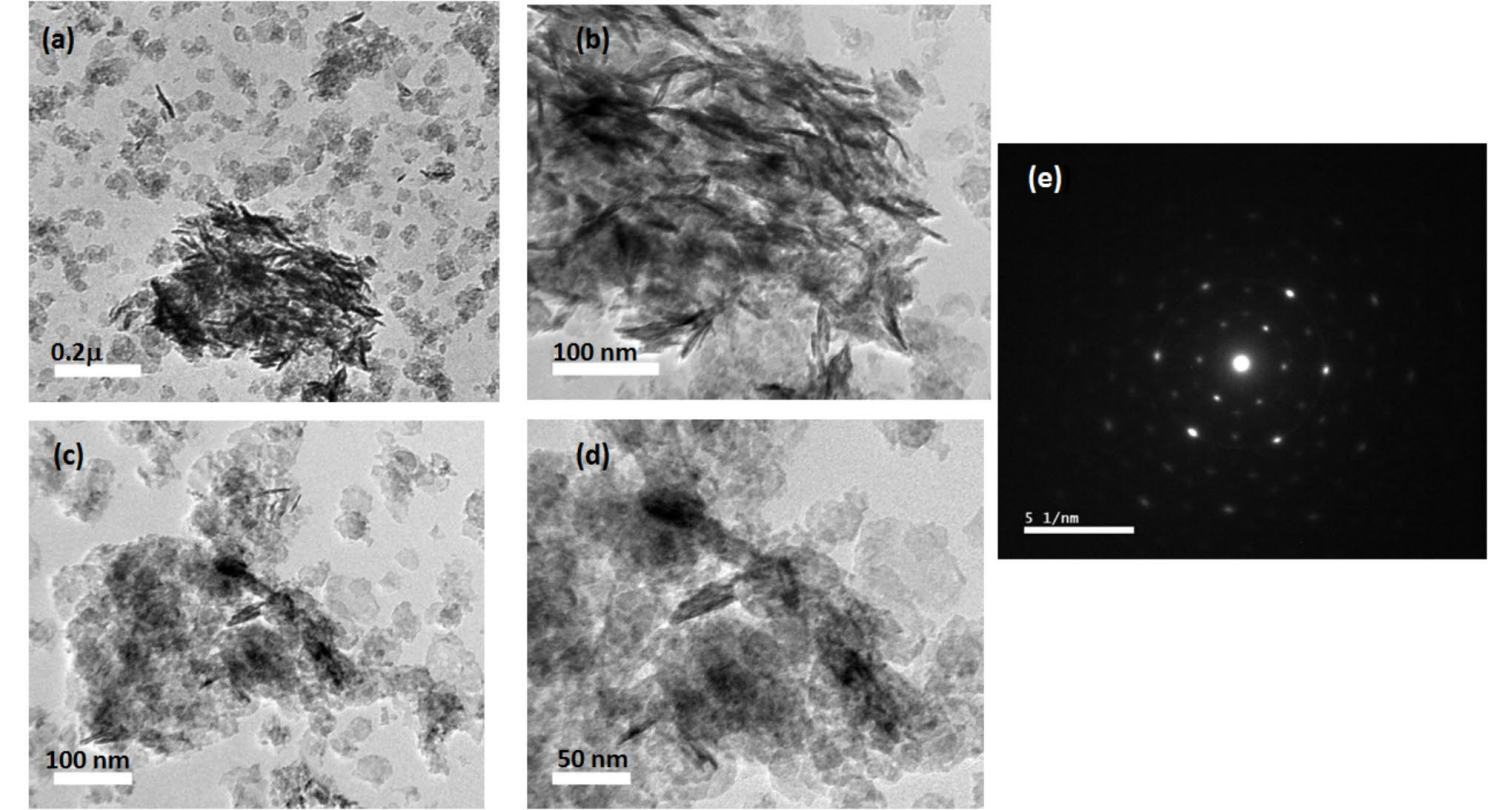
TEM images of NiCo(O).10h, which synthesized at hydrothermal time of 10 h, under different magnifications (**a**, **b**, **c**, **d**), (**e**) showing the corresponding selected area SAED of NiCo(O).10mL.

Figure 6 presents the morphological and structural features of the NiCo(O)10h that were synthesized at the higher hydrothermal time 10 h through TEM and SAED. Figure 6a, b displays a sheet-like/nanoplates structure. On prolonging the reaction time to 10 h, nanosheets with a larger diameter of ~ 75 nm as compared with those of NiCo(O)4h nanosheets, which previously declared above in Fig. 3, were initially formed and then interconnected into each other as depicted in Fig. 6c, d. This implies that the growth process of the nanosheets enlarges up with increasing the hydrothermal time up to 10 h. The sample shows distinct diffraction spots which indicates the crystalline nature of the formed Ni Co (O)10h in Fig. 6e.

### Electrochemical performance

#### Optimization of the electrochemical performance of synthesized CoNi oxides at various pH values of the co-precipitation solution

**Fig. 7:**
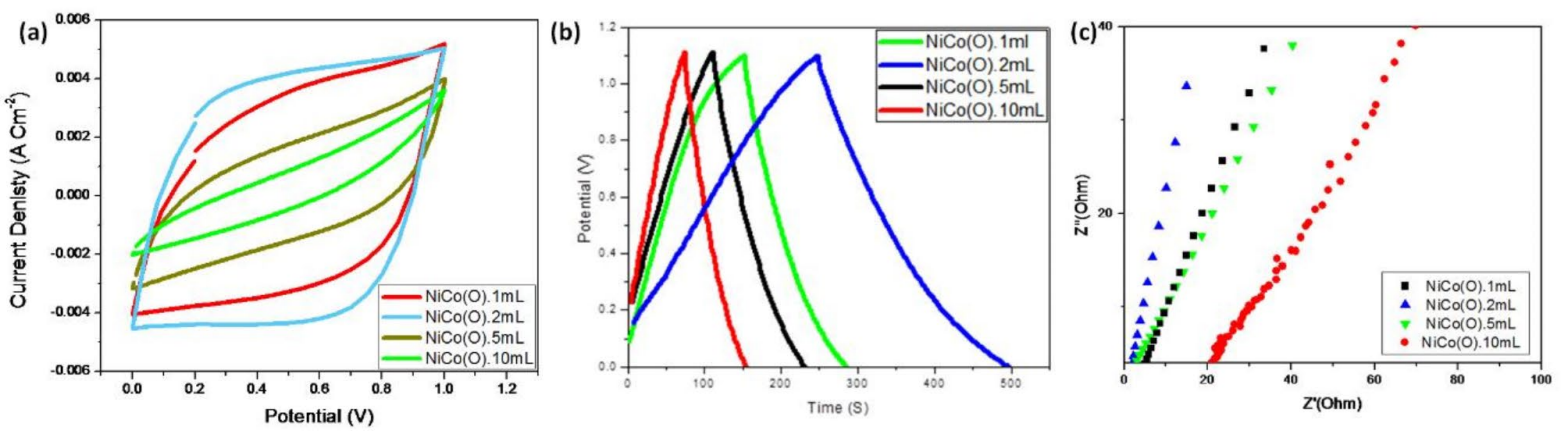
(**a**) CV curves, (**b**) GCD curves at 1 A/g, and (**c**) Nyquist plots of the synthesized Nickel cobalt oxide samples at different added amount of ammonia/different pH values at scan rate 100 mV/s.

*Cyclic Voltammetry measurements, Galvanostatic Charge- Discharge measurements and Electrochemical Impedance Spectroscopy* Electrochemical characteristics of the NiCo(O) samples are analyzed by CV, GCD and EIS curves in 2 M Na_2_SO_4_ electrolyte. In a potential window from 0 V to 1.0 V, CVs of NiCo(O) samples at various scan rates ranging from 5 to 100 mV/s. Different current densities of 0.5, 1.0, 2.0, and 3.0 A/g are pursued for GCD measurements. EIS measurements are carried out between 10^5^ Hz and 0.1 Hz in terms of frequency. Figure 7a shows the CV curves of NiCo(O) prepared using 1, 2, 5 and 10 mL ammonia scanned at 100 mV/s scan rate. Obviously, the CV curves of all the NiCo(O) electrodes exhibit nearly rectangular shapes symmetrical across the zero-current axis, and they do not appear to be oxidation–reduction peaks^50–52^ indicating the typical characteristic of the electrical double layer capacitance that may be attributed to the applied high scan rate during electrochemical measurement ^53–55^. It can be determined that the highest current density and area under the CV curve is obtained for NiCo(O).2mL. This indicates that the NiCo(O).2mL electrode shows the highest specific capacitance among the other NiCo(O) samples synthesized at various added amounts of ammonia/pH values. The NiCo(O).2mL electrode exhibits the best electrochemical energy storage performance compared with the other three samples because it promotes higher electrical conductivity and faster electron transport as it has the highest degree of exfoliation and largest lateral dimension i.e., the highest surface area. It also exhibits a considerably greater CV internal area than the other electrodes, indicating that the interconnected nanoflowers-like nanostructure provide a more surface-active area, and a higher reactive area is helpful for the exchange of ions in the electrodes. Supplementary Figure S7 displays the CVs of NiCo(O).2mL at various scan rates from 5 to 100 mV/s. It is noticed that at high scan rates, the electrolyte ions have not enough time to react with the while electrode active sites and reduce the Faradic reaction^54,56^.

Figure 7b shows the Charge-Discharge curves of NiCo(O) electrodes at fixed current density of 1A/g which are synthesized using different amounts of ammonia, employing Na_2_SO_4_ electrolyte with a potential window of 1 V. Obviously the curves are generally symmetric, indicating the high reversibility of the Faradaic redox reaction. This suggests high electrochemical reversibility and fast reaction kinetics of NiCo(O) oxides electrodes^36,57^. The charging–discharging times revealed that NiCo(O).2mL exhibits the maximum discharging time, which is strongly indicative of the maximum specific capacitance. This is consistent with the observations of CV curves. In addition to the symmetrical nature, a potential platform was observed in charge–discharge curves. This could be due to the charge transfer reaction or electrochemical adsorption/desorption process at the electrode/electrolyte interface^37^. The specific capacitance (Cs), energy and power densities of the NiCo(O) electrodes were calculated using the Eqs. (2), (3) and (4), respectively. The calculated data are presented in Table 3. The highest Csp, E, and P were 253 F g^-1^, 37.8 WhKg^-1^, and 538 WKg^-1^, represented by NiCo(O).2mL. Thus, NiCo(O).2mL is considered the optimum NiCo(O) sample as a supercapacitor electrode out of those synthesized at various pH values of the co-precipitation solution Capacity, thus leading to excellent rate performance.It is obviously noted that NiCo(O).2mL has a double capacitive value of NiCo(O).1mL, despiteNiCo(O).1mL and NiCo(O).2mL have a similar microstructure characteristic as mentioned above; they have the highest degree of the active surface passivation of the crystal growth, the lowest crystal size formed, and the lowest limited degree of aggregation. Whilst, NiCo(O).2mL exclusively depicts their nanosheets have the highest degree of exfoliation and largest lateral dimension i.e., the highest surface area. Consequently, this may provide a clear explanation for the capacitive behavior that was observed.

**Table 3:** Electrochemical parameters of NiCo(O) oxides prepared using different added amounts of ammonia/different pH values.

Samples codes	NiCo(O) .1mL	NiCo(O) .2mL	NiCo(O) .5mL	NiCo(O) .10mL
Specific capacitance (F g^-1^)	120	253	105	70
Energy density (WhKg^-1^)	20	37.8	17.6	11.8
Power density (WKg^-1^)	528	538	519	536

Supplementary Figure S8 illustrates the GCD curves for the NiCo(O).2mL electrode at different current densities (0.5 to 3 A g^-1^) at the potential of 0 to 1 V. Apparently, the GCD curves with symmetrical shape are in accordance with their CV results. Meanwhile, these curves (both GCD and CV) are not extensively distorted from their original shape, even at a high current density, signifying admirable rate capability^58^. In particular, the specific capacitance reaches 167, 253, 154.8 and 94.5 Fg^−1^ at the current density of 0.5, 1, 2 and 3 Ag ^−1^, respectively. Due to the increase of current density, a large number of electrolyte ions will be adsorbed on the surface of electrode, resulting in the rapid decrease of electrolyte ion concentration at the electrode/electrolyte interface. Hence, it will lead to the decrease of specific capacitance with the increasing of current density^59^.

To further analyze the kinetics of ion and charge transfer performance of the electrode materials, electrochemical impedance spectroscopy (EIS) was used. Figure 7c shows the Nyquist plot of the electrodes. The internal resistance (Rs) of the electrode is obtained through the intercept with the X-axis. The Rs values of the NiCo(O).1mL, NiCo(O).2mL, NiCo(O).5mL and NiCo(O).10m Lelectrodes are 5.3 Ω, 1.6 Ω, 3.1Ω and 21.3 Ω, respectively. These results indicate that NiCo(O)0.2mL electrode has the lowest electrochemical reaction resistance (Rs). Nevertheless, NiCo(O).10mL has the highest one, this is a predicted behavior due to their inferior structural features; since it has the lowest degree of the active surface passivation of the crystal growth, the highest crystal size formed, and the highest degree of aggregation.

In the low frequency region, the NiCo(O).2mL, based electrode display near vertical patterns with higher slope than that of other electrodes, indicative of a better capacitive response and low ion transfer resistance^60^. This is resulted due to the special nanostructure morphology and crystallinity of NiCo(O).2mL sample which leads to a great electrochemical performance.

#### Optimization of the electrochemical performance of synthesized CoNi oxides at various hydrothermal time

**Fig. 8:**
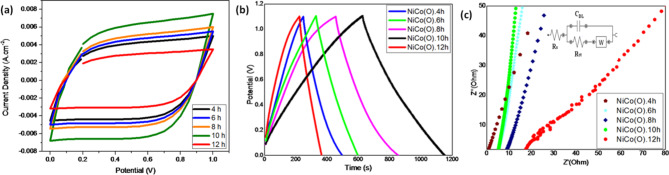
(**a**) Cyclic voltammetry (CV), (**b**) GCD curves and (**c**) Nyquist plots of NiCo(O).2mL oxides at different hydrothermal time at 1 Ag^-1^.

*Cyclic Voltammetry measurements, Galvanostatic Charge–Discharge measurements and Electrochemical Impedance Spectroscopy*: Fig. 8a demonstrates the cyclic voltammetry (CV) curves of NiCo(O).2mL prepared at different hydrothermal time at a scan rate of 100 mV/s. Among them, the sample NiCo(O).2mL as prepared at 10 h had the largest CV area, suggesting the better capacitive performance of this electrode that it could store more energy than others electrodes^61^. The full nano-flower structure composed of interconnected nanoflakes which resulted at 10 h imparts the oxide nanostructure with a larger surface area than the case of a stack of nanoplates over each other’s.

Figure 8b showed the GCD profiles 1 A g^−1^ of prepared NiCo(O).2mL electrodes at different hydrothermal time. It could be found that the charging and discharging parts of GCD profiles were approximately symmetrical, demonstrating excellent coulombic efficiency and low internal resistance during the electrochemical process^62^. The specific capacitance values of NiCo(O).2mL prepared at different hydrothermal time calculated by the discharge parts of GCD profiles were 253, 271.6, 401.32, 525.5 and 138.8 Fg^-1^ for NiCo(O)4h, NiCo(O) 6h, NiCo(O)8h, NiCo(O)10h, and NiCo(O)12h, respectively. The calculated specific capacitances, energy density, and power density are presented in Table 4. Firstly, the capacitance increases gradually by increasing the hydrothermal time from 4 to 6h, then there is a rapid increase as the hydrothermal time elevates up to 8h and 10 h. Finally, sharp downward was recorded as the hydrothermal time increased to 12h.The highest Csp, E, and P were 525 F g^-1^, 88.2 Wh/Kg, and 606 W/Kg, represented by NiCo(O).10h. It can be observed that the NiCo(O)10h electrode has the largest specific capacitance and the NiCo(O)12h has the smallest specific capacitance, which is matched with the results of CV curves. The above results demonstrate that the morphological structure has a significant impact on the electrochemical energy storage performance of the materials. As mentioned above, the rise of hydrothermal time affects the flakes’ growth and dimensionality. Increasing the time lets the hexagonal flakes undergo extensive growth in size/dimensionality during the hydrothermal process. It is clear that the lateral dimension gradually increased as the hydrothermal time increased up to 10 h, and then it significantly decreased in 12 h. Besides, the gradual morphological transformation of the formed binary oxides from exfoliated flake structure as in NiCo(O).4h to semi-flower with a porous structure as in NiCo(O).6h and NiCo(O).8h, and finally to a completed nanoflower with a 3D-porous structure as in NiCo(O) 10h, has a significant impact on the recorded findings. As Pores increase the surface area and active states increase, thus promoting ionic diffusion and permeation^63^. The completed nanoflower architecture that appeared in the oxide sample at NiCo(O)10h with its largest thin flakes and its pore nature led to the greatest Cs, on the other hand, in NiCo(O)12h the sheets become thicker and others bulky have irregular shape which resulted in the least Cs value.

**Table 4:** Electrochemical parameters of NiCo(O).2mL prepared using different hydrothermal time.

Samples Codes	NiCo(O).4h	NiCo(O).6h	NiCo(O).8h	NiCo(O).10h	NiCo(O).12h
Specific capacitance (Fg^-1^)	253	271.6	401.32	525	138.8
Energy density (WhKg^-1^)	37.8	45.3	67.4	88.2	23.32
Power Density (WKg^-1^)	538	601.8	605	606	595.5

To understand the superior rate performance of NiCo(O).2mL prepared at different time, the Nyquist plots of the electrodes are compared in Fig. 8c. No obvious semi-arcs are found in the curves of five samples, indicating small charge transfer impedance. The equivalent series resistances Rs of NiCo(O)4h, NiCo(O)6h, NiCo(O)8h, NiCo(O)10h, and NiCo(O)12h are 1.6, 5,9, 8,9, 5.2 and 16.8 Ω, respectively. The NiCo(O)10h curve is nearly parallel to the y-axis, indicating high capacitive performance. These results indicate that the optimum sample is NiCo(O)10h according to its excellent morphology surface area.

EIS curves are fitted with equivalent circuit using Nova 2.1 software for the optimum sample NiCo(O)10h, as depicted in the inset of Fig. 8c. Rs is the equivalent series resistance comprise resistance of the electrode material, electrolyte, current collectors and contact resistance. Rct is the charge transfer resistance and C_DL_ is the double layer capacitance. In the mid-frequency region of the spectra, a Warburg element (W) describes diffusion processes of the ions through the porous structure of the electrodes. The equivalent circuit parameters obtained for the optimum NiCo(O)10h.electrode presents Rs of 150 m Ω, a low Rct of 3.5 Ω, W of 10.2. mM h and C_DL_ of 612 µF. The large value of C_DL_ indicates the dominant double layer character of NiCo(O)electrode.

To further detect the cycle stability of NiCo(O)10h electrode, 3000 cycles were carried out under 1 A g^-1^ by charging and discharging 1000 times continuously, in Supplementary Figure S9. It can be seen that the capacitance retention rate of NiCo(O)10h electrode is 90.2% due to the nanostructure of the electrode material being damaged with the increase of charge and discharge cycles, which leads to the decrease of activity. The coulomb efficiency is stable during the whole cycle, which indicates that the electrode material has good reversibility and stability^64^. The possible reasons for NiCo(O)10h electrode material which possesses excellent cycling performance are as follows: (i) the synergistic effect between cobalt and nickel provides an active role in redox reaction; (ii) The 3D porous structure of the material gives a larger specific surface area^65–70^, which can add active points for the reaction between electrode/electrolyte^71–73^.

By comparing the NiCo(O) property profile with those published in several articles as demonstrated in Table S3 in Supplementary materials, it is obvious that the optimum sample, that has been prepared in this work, has a modest electrochemical performance as compared with the other’s previous work by employing template/surfactant free, mild and easier synthetic conditions (time, temperature, pH). Briefly, it is concluded that, the well-designed binary nickel cobalt oxides possess improved specific capacities as compared with the monometallic nickel or cobalt oxide. Moreover, electrochemical performance of some researches which employed a soft template or surfactant show better performance than surfactant free ones. Nonetheless, the heavy dosage of pricey soft templates and the diverse post treatment to remove these templates will also pioneer the high-yield synthesis of these Nickel cobalt hydroxides for practical use. Finally, others employed harsh synthetic conditions; temperature up to 700°C, time up to 48 h, and pH up to 14. Thus, the current work satisfies the criteria, since it describes a straightforward and low-cost, economical hydrothermal synthesis method for producing self-assemble flower-like morphology of binary metal oxides with good mechanical performance. It considered a promising candidate for use in supercapacitor devices.

## Conclusions

Self-assemble flower like architecture morphology of binary cobalt nickel oxide has been adapted by controlling the synthesis process parameters including, co-precipitation pH and hydrothermal time by using facile surfactant-free co-precipitation/hydrothermal process. The synthetic process parameters have a significant effect on nanostructure and the morphology characteristics. This occurred through controlling the rate of co-precipitation reaction and the degree of active surface passivation for the crystal growth by changing the former factor, co-precipitation pH value. Followed by increasing the hydrothermal time to modest time, forcing the impact of structural directing growth forward to self-assemble flower-like morphology composed of large interconnected nanosheets. The electrochemical capacitive behavior of the binary metal oxide varies with the sample morphology. The as prepared binary metal oxide which synthesized at co-precipitation pH value of 9 and modest hydrothermal time of 10 h showing an optimum specific capacitance of 525.5 F g^-1^ at a current density of 1 A g^-1^ and energy and power densities of 88.2 WhKg^-1^ and 606 WKg^-1^ respectively, with good cycling stability (90.2% retention after 3000 cycles).

## Electronic supplementary material

Below is the link to the electronic supplementary material.

Supplementary Material 1

## Data Availability

Our manuscript has data included as supplementary materials.
