# Author Correction: Role of synthetic process parameters of nano-sized cobalt/nickel oxide in controlling their structural characteristics and electrochemical energy performance as supercapacitor electrodes

**DOI:** 10.1038/s41598-024-81461-4

**Published:** 2024-12-03

**Authors:** Marwa Adel, Dina Hassan, Marwa A. A. Mohamed, Taher Salah Edin Kassem, Howida Abouel Fetouh, Sara. E. AbdElhafez, Jehan El Nady

**Affiliations:** 1 Fabrication Technology Department, Advanced Technology and New Materials Research Institute, City of Scientific Research and Technological Applications (SRTA-City), New Borg El-Arab, Alexandria, 21934 Egypt; 2 Petroleum Applications Department, Egyptian Petroleum Research Institute (EPRI), Nasr City, 11727 Cairo Egypt; 3 Department of Chemistry, Faculty of Science, Alexandria University, Alexandria, Egypt; 4 Electronic Materials Department, Advanced Technology and New Materials Research Institute, City of Scientific Research and Technological Applications (SRTA-City), New Borg El-Arab, Alexandria, 21934 Egypt

Correction to: *Scientific Reports* 10.1038/s41598-024-77180-5, published online 08 November 2024

In the original version of this Article, Figure 8 was incorrect. The original Figure 8 and accompanying legend appear below.

**Fig. 8:**
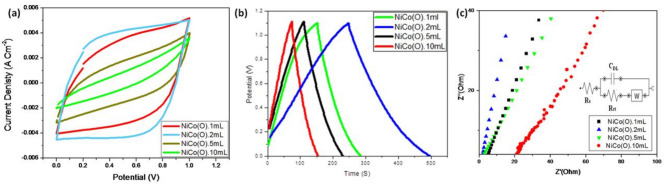
(**a**) Cyclic voltammetry (CV), (**b**) GCD curves and (**c**) Nyquist plots of NiCo(O). 2mL oxides at different hydrothermal time at 1 Ag^−1^.

The original Article has been corrected.

