# Supplementary material for: Role of synthetic process parameters of nano-sized cobalt/nickel oxide in controlling their structural characteristics and electrochemical energy performance as supercapacitor electrodes
Source: Sci Rep. 2024 Nov 8;14:27187. doi: 10.1038/s41598-024-77180-5 (PMC11549468; doi:10.1038/s41598-024-77180-5)
Supplement: Supplementary file 1 — Supplementary Material 1 [file 41598_2024_77180_MOESM1_ESM.docx]

***Supplementary Figure* S1**: EDX Spectrum of samples

- Effect of ***Synthesis process co-precipitation pH***

NiCo(O).1Am

NiCo(O).2Am

NiCo(O).5Am

NiCo(O).10Am

***Supplementary Figure*** S1a. EDX Spectrum of of synthesized Nickel cobalt oxide samples at different added amount of ammonia/different pH values

***Table S1*. Elemental composition of NiCo(O) samples prepared at different added amounts of ammonia/different pH values by EDX analysis**

| Samples codes  Element | NiCo(O).1mL | | NiCo(O).2mL | | NiCo(O).5mL | | NiCo(O).10mL | |
| --- | --- | --- | --- | --- | --- | --- | --- | --- |
|  | **At%** | **Mass%** | **At%** | **Mass%** | **At%** | **Mass%** | **At%** | **Mass%** |
| Co | 45.24 | 47.9075 | 42.49 | 47.2994 | 52.87 | 57.5341 | 57.38 | 61.5044 |
| Ni | 43.77 | 47.8134 | 39.38 | 45.2114 | 32.56 | 36.5488 | 30.40 | 33.6017 |
| O | 11.00 | 4.2791 | 18.13 | 7.4892 | 14.57 | 5.9171 | 12.22 | 4.8938 |
| Total | **100** | **100** | **100** | **100** | **100** | **100** | **100** | **100** |

- Effect of ***Synthesis process hydrothermal time***

NiCo(O).4h

NiCo(O).6h


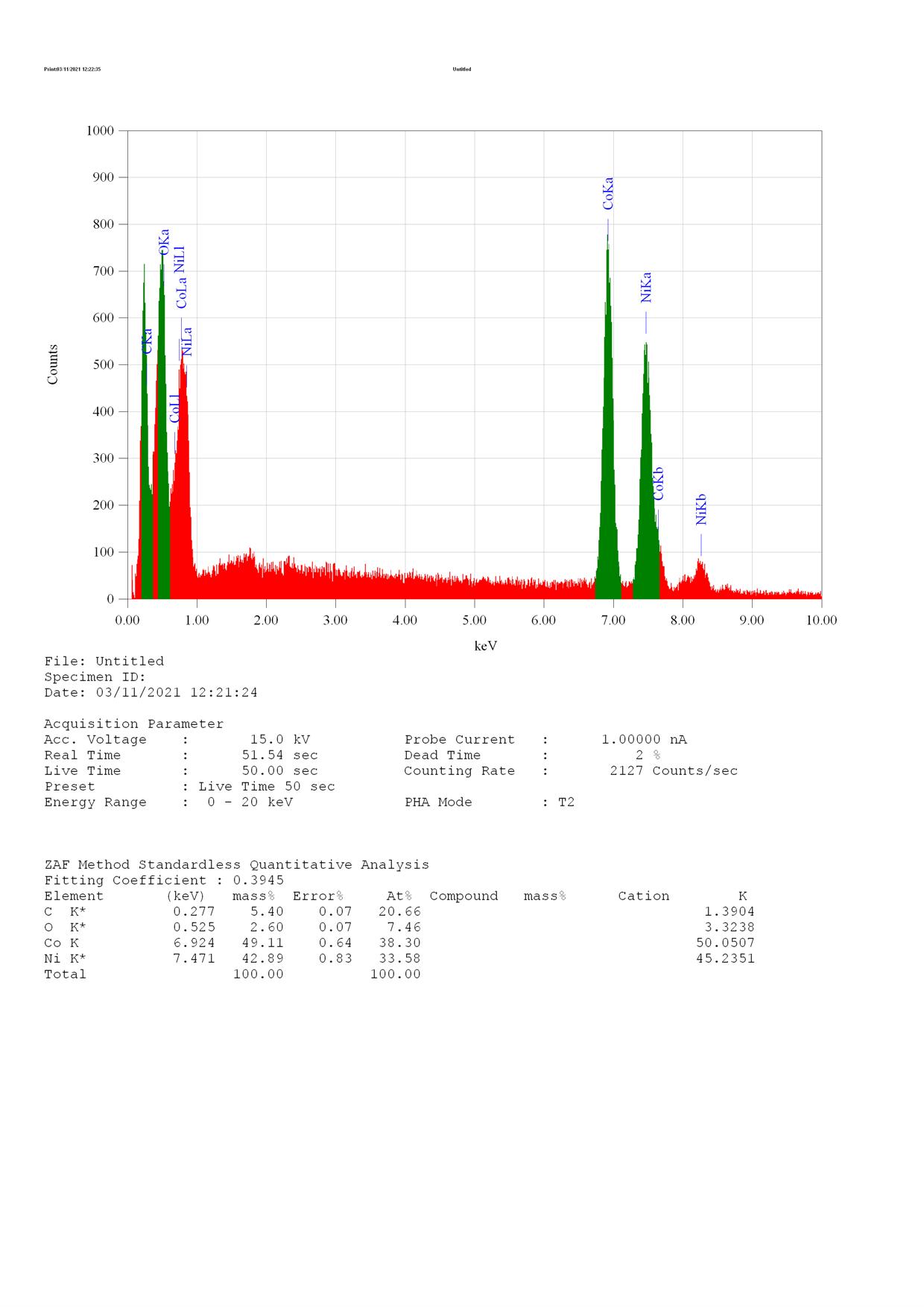


NiCo(O).10h

NiCo(O).8h


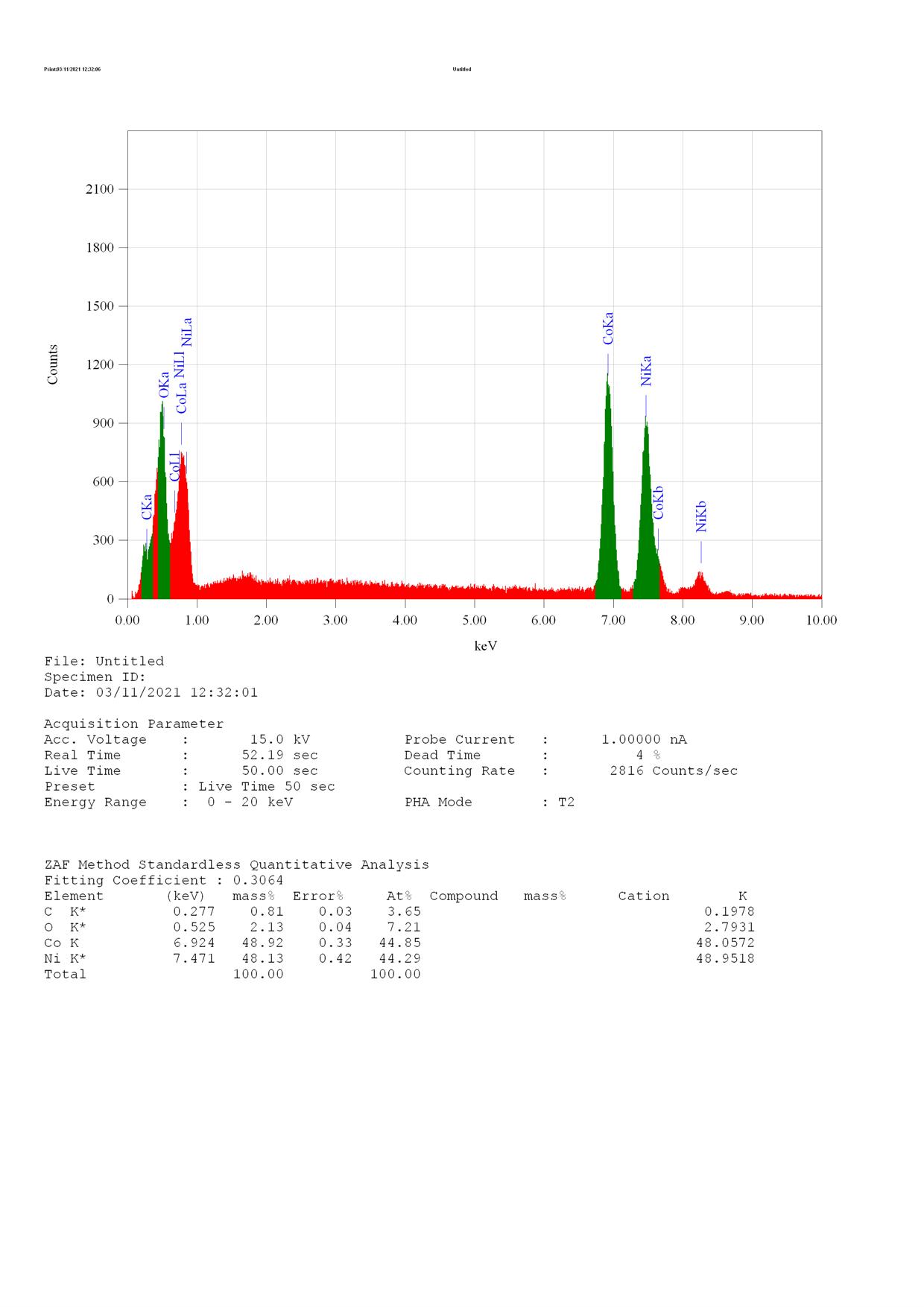


NiCo(O).12h

***Supplementary Figure*** S1b. EDX Spectrum of synthesized Nickel cobalt oxide samples at different hydrothermal time

***Table S2.* Elemental composition of NiCo(O) samples prepared at different hydrothermal time by EDX analysis**

| Samples codes  Element | NiCo(O).4h | | NiCo(O).6h | | NiCo(O).8h | | NiCo(O).10h | | NiCo(O).12h | |
| --- | --- | --- | --- | --- | --- | --- | --- | --- | --- | --- |
|  | **At%** | **Mass%** | **At%** | **Mass%** | **At%** | **Mass%** | **At%** | **Mass%** | **At%** | **Mass%** |
| Co | 42.49 | 47.29 | 40.2 | 46.328 | 38.3 | 50.05 | 45.39 | 48.405 | 44.8 | 48.06 |
| Ni | 39.38 | 45.21 | 36.9 | 43.789 | 33.6 | 45.24 | 42.63 | 46.892 | 44.2 | 48.95 |
| O | 18.13 | 7.489 | 22.9 | 9.8825 | 28.1 | 4.71 | 11.98 | 4.7025 | 10.8 | 2.99 |
| Total | **100** | **100** | **100** | **100** | **100** | **100** | **100** | **100** | **100** | **100** |

***Supplementary Figure* S2 (XRD spectra of Nickel cobalt hydroxide samples)**


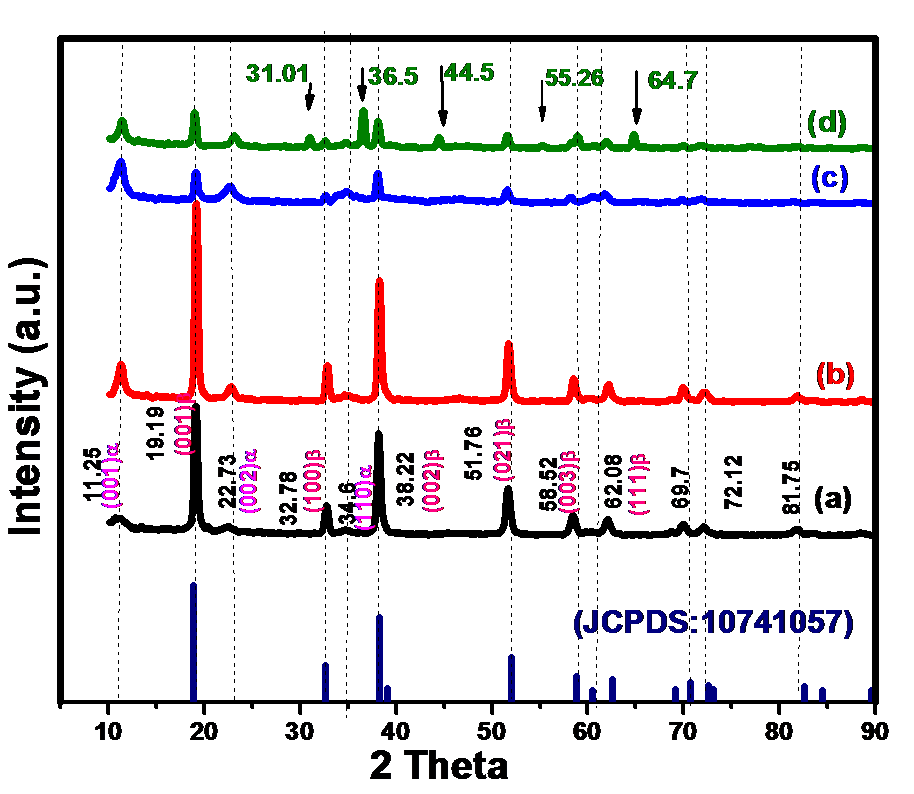


***Supplementary Figure S2:*** XRD spectra of synthesized Nickel cobalt hydroxide samples at different added amount of ammonia/different pH values a) (NiCo(OH).1mL, b) NiCo(OH).2mL, c) NiCo(OH).5mL and d) NiCo(OH).10mL.

***Supplementary Figure* S3 (SEM of Nickel cobalt hydroxide samples)**


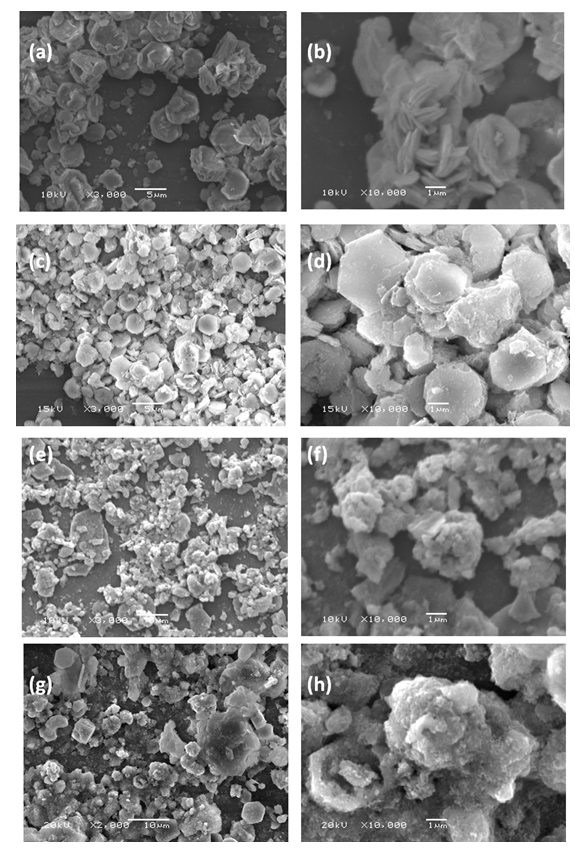


***Supplementary Figure S3.*** The SEM micrographs of (a) NiCo(OH).1mL, (b) Magnified micrograph of NiCo(OH).1mL, (c) NiCo(OH).2mL, (d) Magnified micrograph of NiCo(OH).2mL, (e) NiCo(OH).5mL, (f) Magnified micrograph of NiCo(OH).5mL, (g) NiCo(OH).10mL and (h) Magnified micrograph of NiCo(OH).10mL

***Supplementary Figure* S4 (HR-TEM)**

**
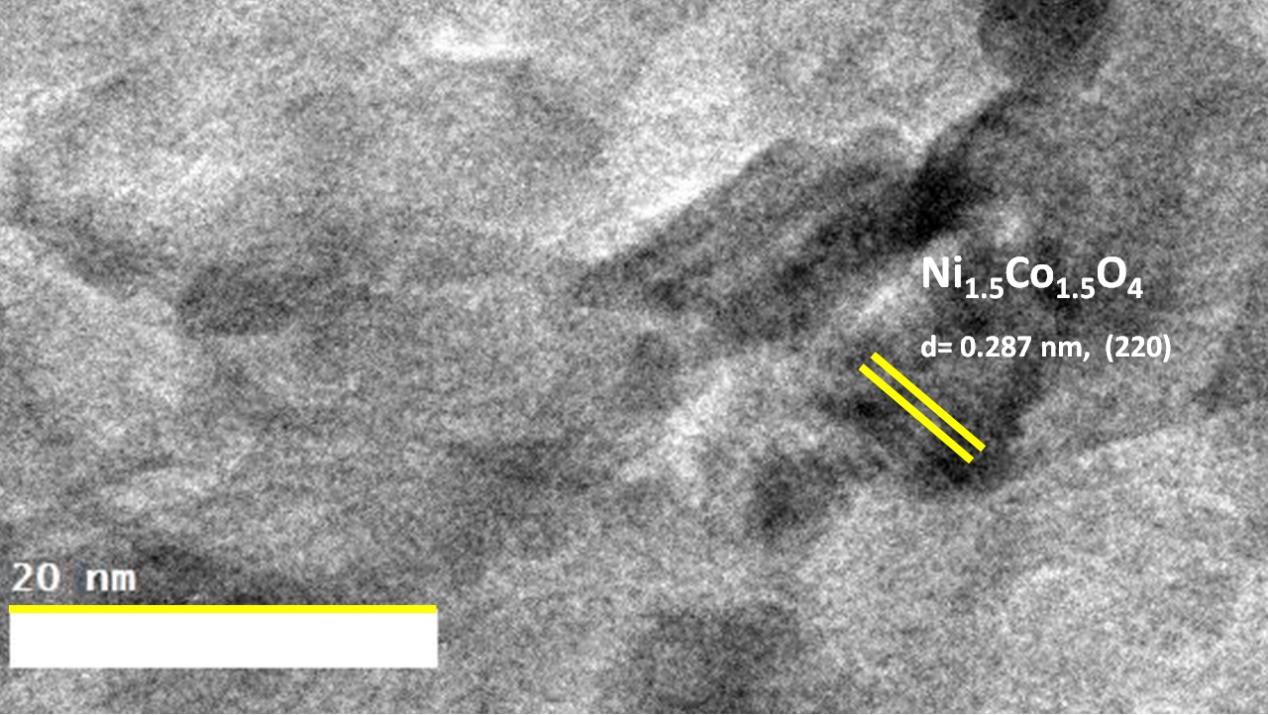
**

**(a)**


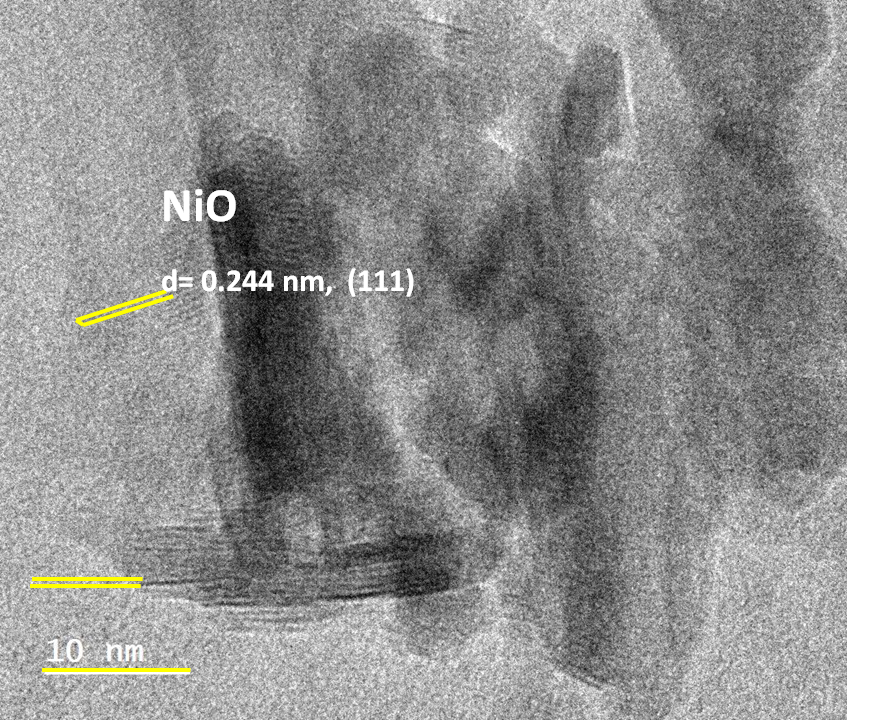


**(b)**


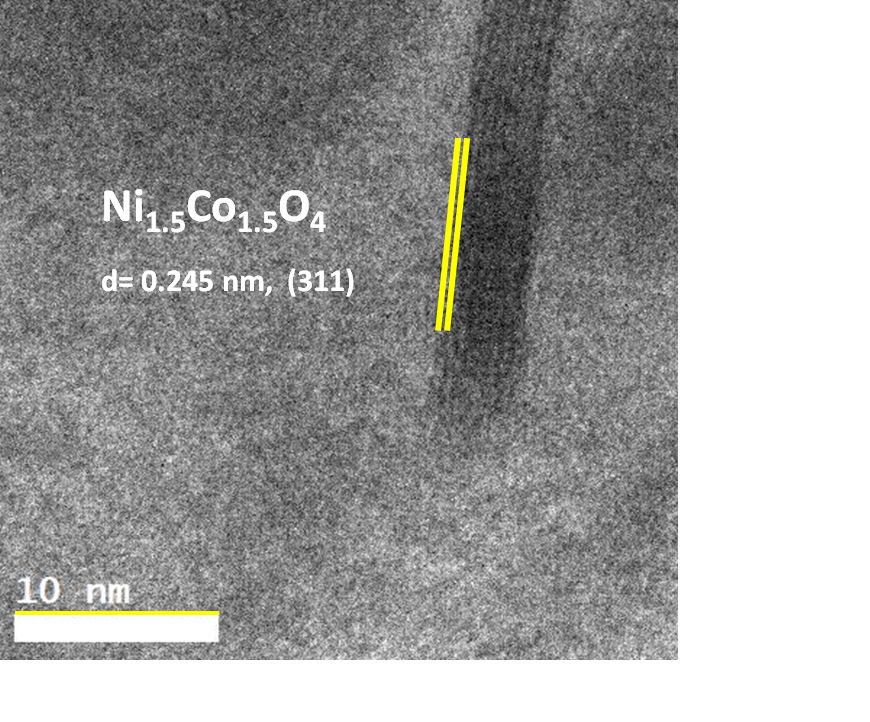


**(c)**

***Supplementary Figure S4*.** (a,b) HR-TEM images of NiCo(O).2mL, under different magnifications, and (c) HR-TEM images of NiCo(O).10mL

***Supplementary Figure* S5 (XRD Spectra of Nickel cobalt hydroxide samples at different hydrothermal time)**

**
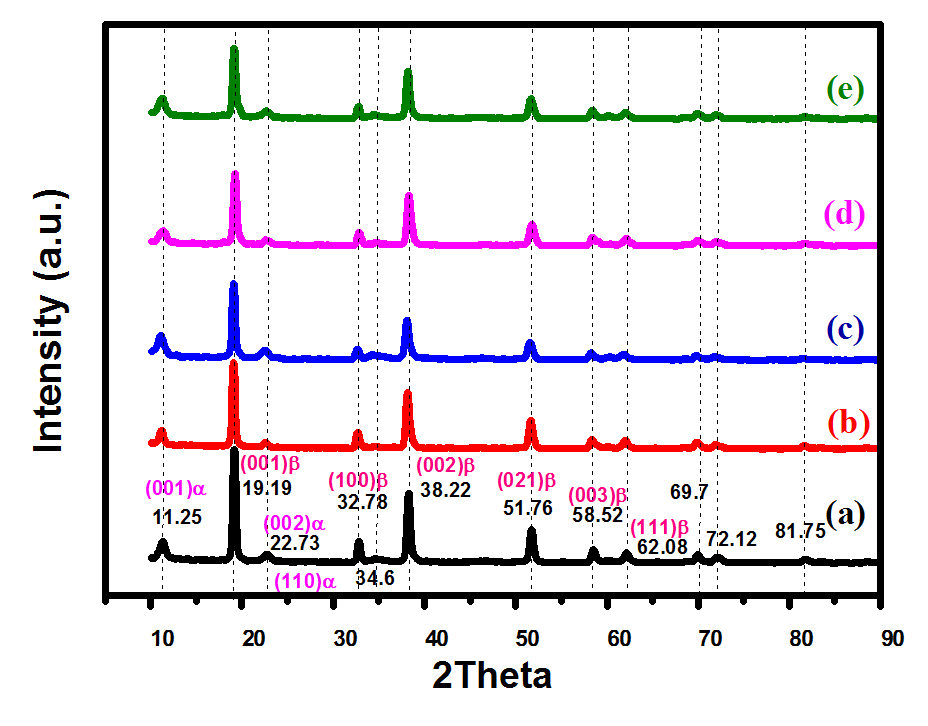
**

***Supplementary Figure S5.*** XRD spectra of synthesized Nickel cobalt hydroxide samples at different hydrothermal time a) (NiCo(OH).4h, b) NiCo(OH).6h, c) NiCo(OH).8h, d) NiCo(OH).10h) and e) NiCo(OH).12h).

***Supplementary Figure* S6 SEM of the synthesized Nickel cobalt hydroxide samples at different hydrothermal time**

**
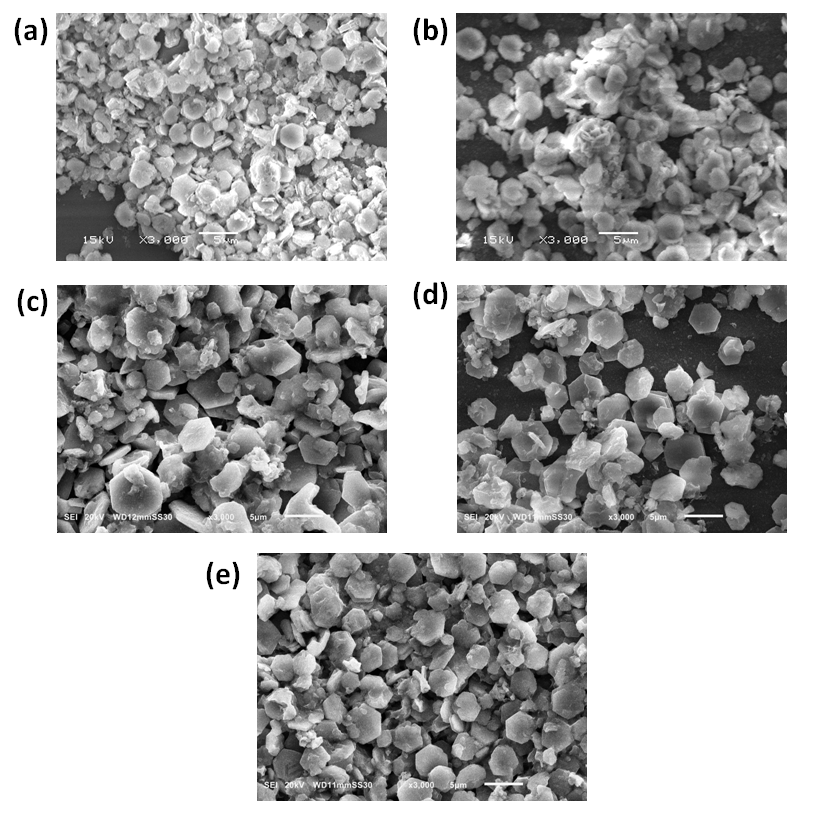
**

***Supplementary Figure S6.*** Micrographs of the synthesized Nickel cobalt hydroxide samples at different hydrothermal time a) (NiCo(OH).4h, b) NiCo(OH).6h, c) NiCo(OH).8h, d) NiCo(OH).10h) and e) NiCo(OH).12h).

***Supplementary Figure* S7 CV curves of NiCo(O).2mL sample at different scan rates.**

**
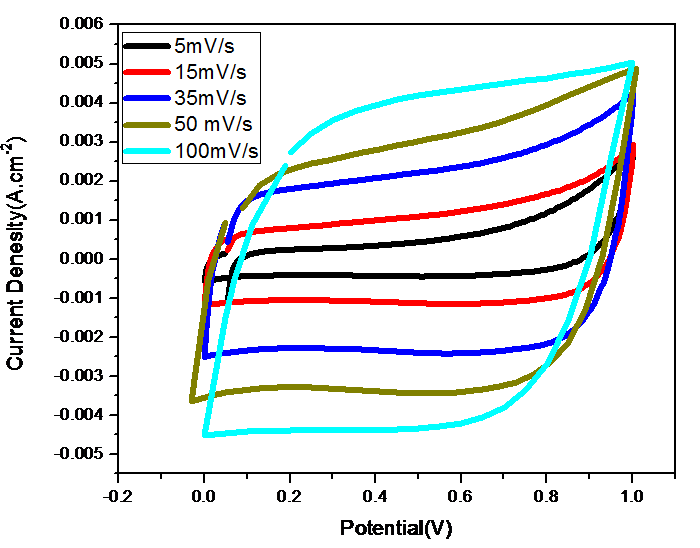
**

***Supplementary Figure S7*.** CV curves of NiCo(O).2mL sample at different scan rates.

***Supplementary Figure* S8** **(GCD curves for NiCo(O).2mL sample at different current densities)**

**
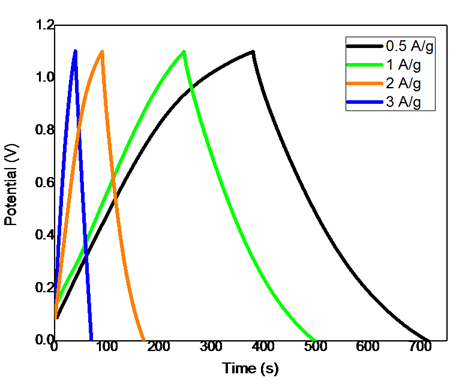
**

***Supplementary Figure S8.*** GCD curves for NiCo(O).2mL sample at different current densities

***Supplementary Figure S9 (*The cycling performance of NiCo(O)10h at the current density of 1 A g^-1^*)***


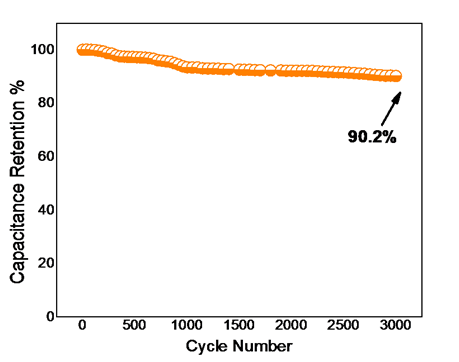


***Supplementary Figure S9.*** The cycling performance of NiCo(O)10h at the current density of 1 A g^-1^

**The property profile of mono or bimetallic NiCo(O)reported in some papers** is listed in Table S3

***Table S3.* The property profile of mono or bimetallic NiCo(O)reported in some papers**

| Method | Morphology | Surfactant/soft template | Testing conditions | Electrochemical performance | Reference |
| --- | --- | --- | --- | --- | --- |
| Hydrothermal followed by heat treatment | NiO: Nanoslices  : Nanoplates  : Nanocolumns | surfactant free | 160 °C for 8 h  400 °C for  about 2 h.  pH 14 | - 176 F/g) - (285 F/g) - (390 F/g) at 5 A/g. | Zhang. X. et al. (14)  (2010) |
| Hydrothermal followed by heat treatment | (Co_3_O_4_): needle-like nanorods | surfactant free | 220 °C for 18 h.  300°C for 2h | - 111 F g^-1^ | Zhu. T. et al (20)(2010) |
| Co-precipitation followed by heat treatment | Bimetallic NiCo_2_O_4_ oblong-shaped nanoparticles | surfactant free | 400°C for 4 h  Annealing temperature 700 °C for 4h | - 249.8 F g^-1^ at 0.5Ag^-1^ | Chang K et al. (74)  (2015) |
| Hydrothermal followed by heat treatment | Bimetallic NiCo_2_O_4_ 3D flower-like morphology | Hexamethylene tetraamine as  nanostructure growth assisting agent | Hydrothermal temperature 120°C for 2h  Annealing temperature 500 °C for 2h | - optimum specific capacitance   of 750 F/g at 1 Ag^-1^ | Zhang. J. et al. (34)(2015) |
| Hydrothermal method followed by heat treatment | Bimetallic NiCo_2_O_4_ sea urchin-like morphology | surfactant free | 100 °C for 48h.  300 °C for 3 h | - 658 F g^-1^ at a current density of 1 A g^-1^. | Xiao J. et al. (35)(2011) |
| Solvothermal | - Bimetallic 1D porous NiCo_2_O_4_ nanorods | surfactant free | 160 °C for 12 h.  Annealing temperature 350 °C for2 h | - 417.1 F g^−1^ at 1 A g^−1^ | Dang S.et al (75) (2019) |
| Hydrothermal | - Bimetallic NiCo_2_O_4_ flower-like morphology | hexamethylenetetramine (HMTA) | 90°C for 6h.  300 °C for 2 h | - 776 F g^−1^at a current density of 1 A g-1 | Chen Y.et al (76) (2019) |
| Hydrothermal | - NiO sphere-like morphology - NixCo3-xO4(I),dandelion-like nanowires - NixCo3-xO4(II) nanorods where x = 2.3, 1.6, | surfactant free | 150 °C for 10h  Annealing temperature 500 ◦C for 2h | - 291F g^-1^ - 590F g^-1^ - 1948 F g^-1^ | Ahmad R. et al (77) (2023) |
| Co-precipitation/ hydrothermal  process followed by heat treatment | Bimetallic NiCo_2_O_4_ 3D-nanoflower-like morphology | surfactant-free | 120°C for 10h  Annealing temperature 300 °C for 2 h  pH 9 | - 525.5 F g^-1^ at a current density of 1 A g^-1^ - energy 88.2 WhKg^-1^ - power density of 606 WKg^-1^ | This work |
